# Coronary Arterial Anomalies in Adults with Congenital Heart Disease: From Detection to Management Strategies

**DOI:** 10.3390/jcdd13090444

**Published:** 2026-09-07

**Authors:** Ahmed Wahba, Sameh M. Said

**Affiliations:** 1 Division of Pediatric and Adult Congenital Cardiac Surgery, Maria Fareri Children’s Hospital, Department of Surgery, Westchester Medical Center, New York, NY 10595, USA; ahmdasamh355@gmail.com; 2 Department of Surgery, New York Medical College, New York, NY 10595, USA

**Keywords:** adult congenital heart disease, coronary artery anomalies, anomalous aortic origin of coronary artery, arterial switch operation, post-Ross, coronary CT angiography, cardiac magnetic resonance, sudden cardiac death

## Abstract

Congenital coronary artery anomalies, whether native, associated with structural heart defects, or acquired following surgical repair, are an increasingly recognized cause of morbidity and sudden cardiac death in the expanding adult congenital heart disease (ACHD) population. Their clinical significance is heightened as the first generation of arterial switch operation (ASO) recipients reaches adulthood. This review presents a practical, evidence-based framework for the classification, multimodality imaging evaluation, risk stratification, and surgical and interventional management of coronary anomalies across the ACHD spectrum. Recommendations from the 2025 ACC/AHA and 2020 ESC guidelines are synthesized, key divergences in the approach to asymptomatic patients are highlighted, and critical knowledge gaps, including emerging technologies and the uncertain long-term coronary outcomes of the ASO generation, are discussed.

## 1. Introduction

Congenital heart disease (CHD) is the most common major birth defect, affecting approximately 1% of live births worldwide [1,2,3]. Extraordinary advances in pediatric cardiac surgery and interventional cardiology over the past five decades have transformed the natural history of CHD, with more than 90% of affected infants now surviving into adulthood [1,4]. The adult congenital heart disease (ACHD) population has grown rapidly, with an estimated 1.4 million adults living with CHD in the United States as of 2010, and adults now accounting for approximately two-thirds of the total CHD population [1,4,5]. This population continues to expand at an estimated rate of 5% annually [5].

Within this growing population, congenital coronary artery (CA) anomalies represent an important but underrecognized source of morbidity and mortality. Coronary anomalies in the ACHD population are uniquely complex, encompassing three distinct categories: (1) isolated congenital coronary anomalies that may be incidentally discovered or present with ischemic symptoms in adulthood; (2) coronary anomalies intrinsically associated with structural congenital heart defects, such as the anomalous left anterior descending (LAD) artery crossing the right ventricular outflow tract (RVOT) in tetralogy of Fallot (TOF); and (3) acquired coronary complications resulting from prior cardiac surgical repair, most notably after the arterial switch operation, Ross procedure, and repair of anomalous left coronary artery from the pulmonary artery (ALCAPA) [5,6,7,8].

CA anomalies are the second most common cardiovascular cause of sudden cardiac death (SCD) in young athletes after hypertrophic cardiomyopathy, and they account for approximately 15% of exercise-related sudden deaths [5,9]. In autopsy series, the overall prevalence of anomalous aortic origin of a coronary artery (AAOCA) ranges from 0.2% to 0.5%, while clinical imaging series report prevalences ranging from less than 1% to more than 5% [7]. The clinical significance of these anomalies varies widely, from entirely benign variants requiring no intervention to life-threatening conditions demanding urgent surgical repair.

The purpose of this review is to provide a comprehensive, clinically oriented framework for the detection, risk stratification, and management of CA anomalies across the full spectrum of ACHD. This narrative review was conducted through a systematic search of the literature aiming to comprehensively examine coronary artery anomalies in adults with congenital heart disease (ACHD), with a focus on isolated congenital anomalies, postoperative coronary complications, diagnostic modalities, and management strategies.

We searched PubMed, Embase, and the Cochrane Library databases from January 2008 to June 2024 using combinations of keywords including “coronary artery anomalies,” “adult congenital heart disease,” “arterial switch operation,” “ALCAPA,” “Ross procedure,” “postoperative coronary complications,” and “diagnostic imaging.” We additionally reviewed references of relevant articles to identify further pertinent studies.

Inclusion criteria comprised original clinical studies, meta-analyses, guidelines, consensus statements, and high-quality expert reviews addressing coronary anomalies in ACHD adults. Exclusion criteria were non-English publications, pediatric-only studies without adult data, and case reports except when illustrating rare surgical complications.

Relevant articles were screened for quality and clinical relevance. Where strong evidence from prospective or registry data was unavailable due to the rarity of certain anomalies, we included expert consensus and opinion from established authorities in the field.

This approach enables a broad, yet critically appraised, synthesis of current knowledge and clinical practice pertinent to this heterogeneous and complex patient population.

This review also integrates the most recent guideline recommendations, including the 2025 ACC/AHA/HRS/ISACHD/SCAI ACHD Guideline and the 2025 ACR Appropriateness Criteria for CA anomalies, with contemporary evidence from surgical series, imaging studies, and long-term outcome data [8,10].

## 2. Classification of Isolated Coronary Artery Anomalies

### 2.1. Historical Classification Systems

Several classification systems have been proposed for congenital CA anomalies. The classification by Greenberg et al. categorizes anomalies based on origin, course, and termination [11]. Angelini proposed a comprehensive system distinguishing anomalies of origin and course, intrinsic coronary anatomy, and termination, further subdividing them into hemodynamically significant and insignificant variants [7]. The European Society of Cardiology Development, Anatomy, and Pathology Working Group published a position statement in 2016 providing an embryology-based classification framework that integrates developmental mechanisms with clinical phenotypes.

For clinical purposes, a modified classification system (Table 1 and Table 2) organized around three major categories provides the most practical framework for the ACHD clinician [12].

### 2.2. Practical Classification

1. Anomalies of Origin and Course

**Figure 1:**
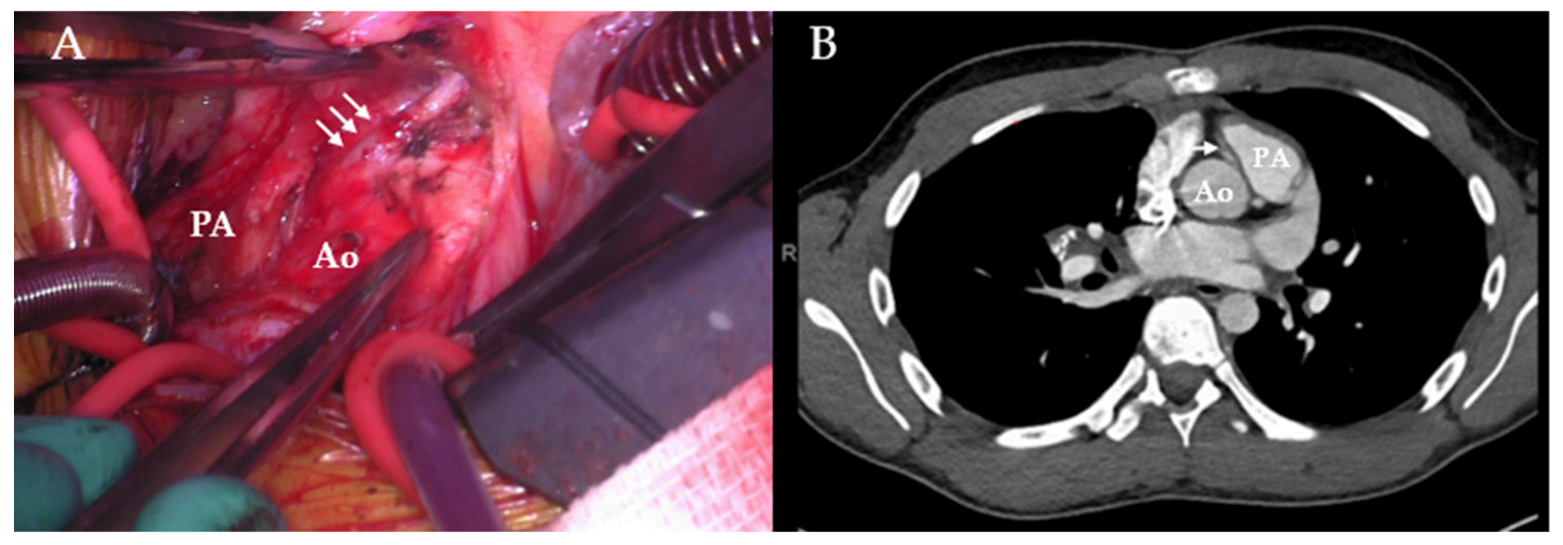
(**A**) intraoperative photo showing the right coronary artery (multiple white arrows) running an inter-arterial course between the aorta and the main pulmonary artery, (**B**) preoperative computed tomography scan demonstrating the same course. In this case, there is no intramural segment; (Ao: ascending aorta; PA: pulmonary artery).

**Figure 2:**
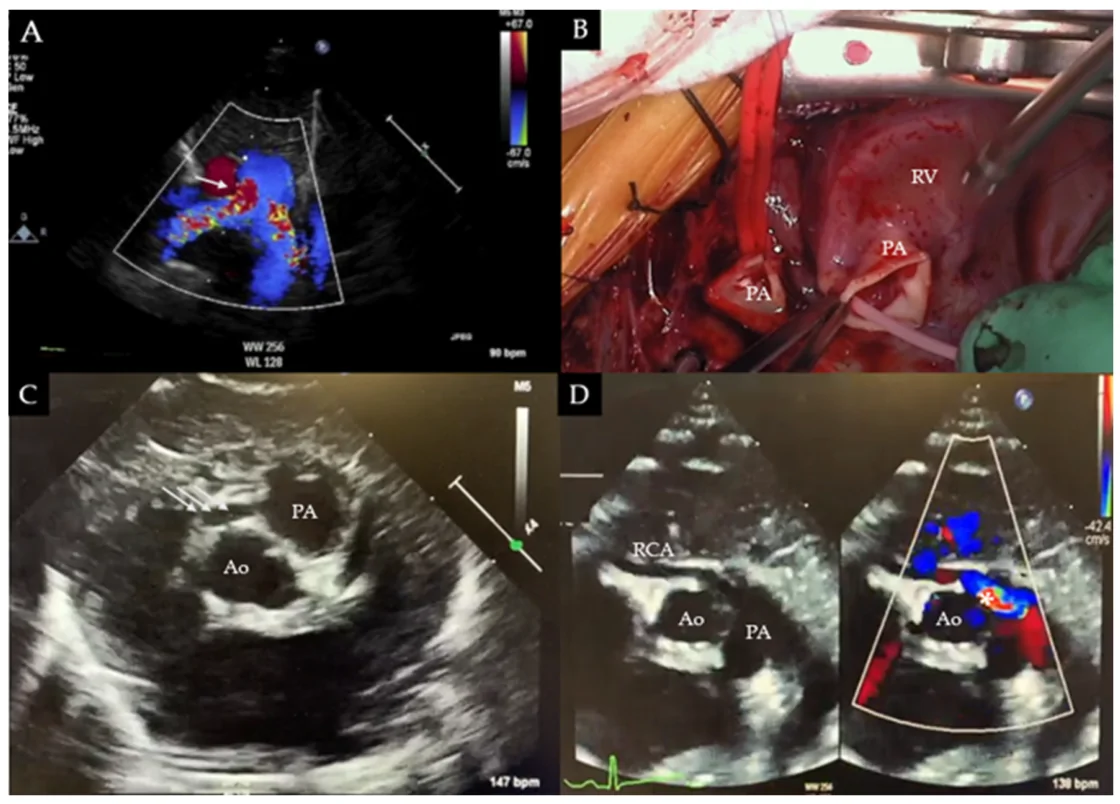
(**A**–**D**): Anomalous origin of the coronary artery from the pulmonary artery, (**A**) preoperative transthoracic echocardiography showing retrograde flow (white arrow) in the main pulmonary artery which is diagnostic, (**B**) intraoperative photo showing the transected main pulmonary artery (PA) and a probe is inserted in the left coronary artery ostium that is arising from the pulmonary root (ALCAPA), (**C**) transthoracic echocardiography showing the anomalous right coronary artery arising (multiple white arrows) from the main pulmonary artery (ARCAPA), and (**D**) retrograde flow (white asterisk) is seen into the pulmonary artery; RV: right ventricle; PA: pulmonary artery; Ao: aorta; RCA: right coronary artery.

**Figure 3:**
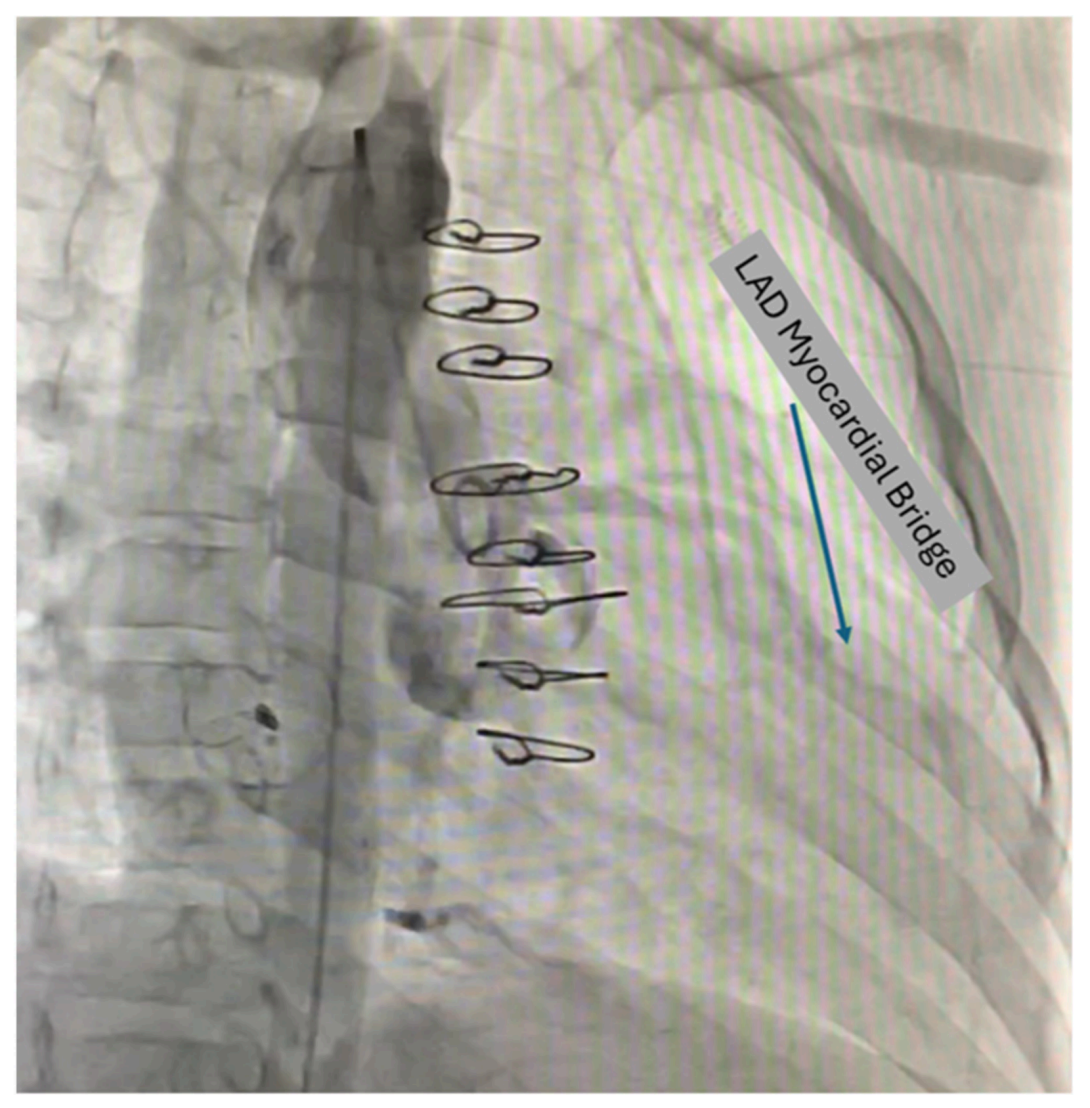
Cardiac catheterization showing a deep and long myocardial bridge (arrow) affecting the left anterior descending coronary artery; LAD: left anterior descending coronary artery.

**Figure 4:**
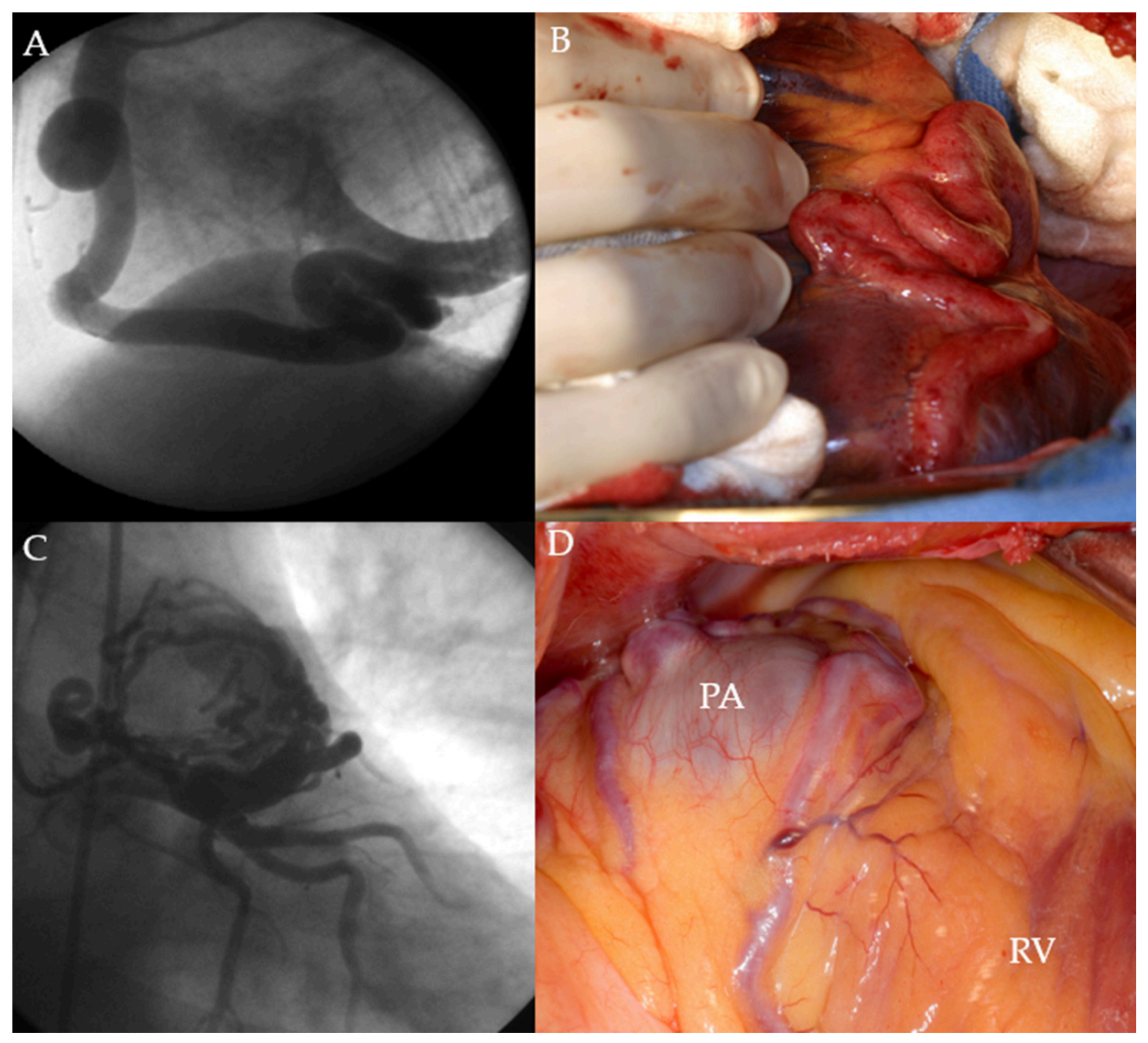
(**A**–**D**): Coronary artery fistulas represent abnormal communication between a coronary artery and a cardiac chamber, coronary sinus (**A**,**B**), superior vena cava or pulmonary artery (**C**,**D**). (**A**,**C**) represents cardiac catheterization images, while (**B**,**D**) are intraoperative photos; PA: pulmonary artery; RV: right ventricle.

This category encompasses the most clinically significant coronary anomalies and includes:

- Anomalous aortic origin of a coronary artery (AAOCA) from the wrong sinus of Valsalva: The most clinically important anomaly, with four recognized patterns: left main coronary artery from the right sinus, right coronary artery from the left sinus, circumflex or left anterior descending artery from the right sinus, and origin from the noncoronary sinus. The proximal course of the anomalous vessel determines clinical significance, with four described pathways: interarterial (between the aorta and pulmonary artery) (Figure 1A,B), retroaortic, prepulmonic, and trans-septal (subpulmonic) [13]. The interarterial course carries the highest risk of myocardial ischemia and sudden death [5,6,7,9].
- Anomalous origin of a coronary artery from the pulmonary artery (ALCAPA (Figure 2A,B)/ARCAPA (Figure 2C,D)): ALCAPA occurs in approximately 1 in 300,000 live births and is almost universally fatal in infancy without surgical correction. A small subset of patients with well-developed collateral circulation (the “adult type”) survive into adulthood, where they may present with dyspnea, angina, heart failure, ventricular arrhythmias, or sudden cardiac death [14]. A systematic review of 279 adult ALCAPA cases reported over 60 years found that the majority were women, dyspnea was the most common symptom, and the mean age at surgery was 37.5 years [9,15,16].
- Single coronary artery: A rare anomaly (0.024–0.066% prevalence) in which the entire myocardium is supplied by a single vessel arising from a single ostium [12]. Clinical significance depends on the course of the branches, particularly whether any segment traverses between the great vessels [7].
- High takeoff and anomalous ostial location: Generally benign but clinically important during cardiac surgery, aortic cannulation, and coronary angiography [12].

2. Anomalies of Intrinsic Coronary Anatomy

- Myocardial bridging: A band of myocardial muscle overlying a coronary artery segment, most commonly the mid-left anterior descending artery. Prevalence ranges from 0.5–2.5% at angiography to 15–85% at autopsy [12]. Although usually benign, deep bridges can cause symptomatic ischemia, particularly in the setting of left ventricular hypertrophy or hypertrophic cardiomyopathy (Figure 3).
- Congenital ostial stenosis and coronary ectasia: Ostial stenosis may occur as an isolated finding or in association with supravalvar aortic stenosis (Williams syndrome), where coronary ectasia is characteristic [12].

3. Anomalies of Termination

- Coronary artery fistulae: Abnormal communications between a coronary artery and a cardiac chamber, coronary sinus (Figure 4A,B), superior vena cava, or pulmonary artery (Figure 4C,D). The incidence is 0.1–0.2% in patients undergoing coronary angiography. The right coronary artery is most commonly involved (60%), and the most frequent drainage sites are the right ventricle (39%), right atrium (33%), and pulmonary artery (20%) [12]. Fistulae may be congenital or acquired and are an important component of pulmonary atresia with an intact ventricular septum [6,8].

4. Hemodynamically Significant Versus Benign Anomalies

A practical clinical distinction separates hemodynamically significant anomalies, characterized by abnormalities of myocardial perfusion with increased risk of ischemia or sudden death, from hemodynamically insignificant variants [12]. Hemodynamically significant anomalies include AAOCA with an interarterial course, ALCAPA/ARCAPA, large coronary fistulae with significant shunting, and symptomatic deep myocardial bridging. Benign variants include retro-aortic circumflex artery, separate ostia of the left anterior descending and circumflex arteries, and high takeoff without associated pathology [5,6].

## 3. Coronary Anomalies Associated with Specific Congenital Heart Defects

The association between CA anomalies and structural congenital heart defects CHD is well established and carries profound implications for surgical planning, reoperation risk, and long-term surveillance. Pre-operative evaluation with computed tomographic angiography (CTA) or cardiac magnetic resonance (CMR) imaging is recommended for all patients with previously palliated or repaired conotruncal anomalies, conduits, and prior aortic root surgery (Table 3) [12,17,18].

### 3.1. Tetralogy of Fallot

**Figure 5:**
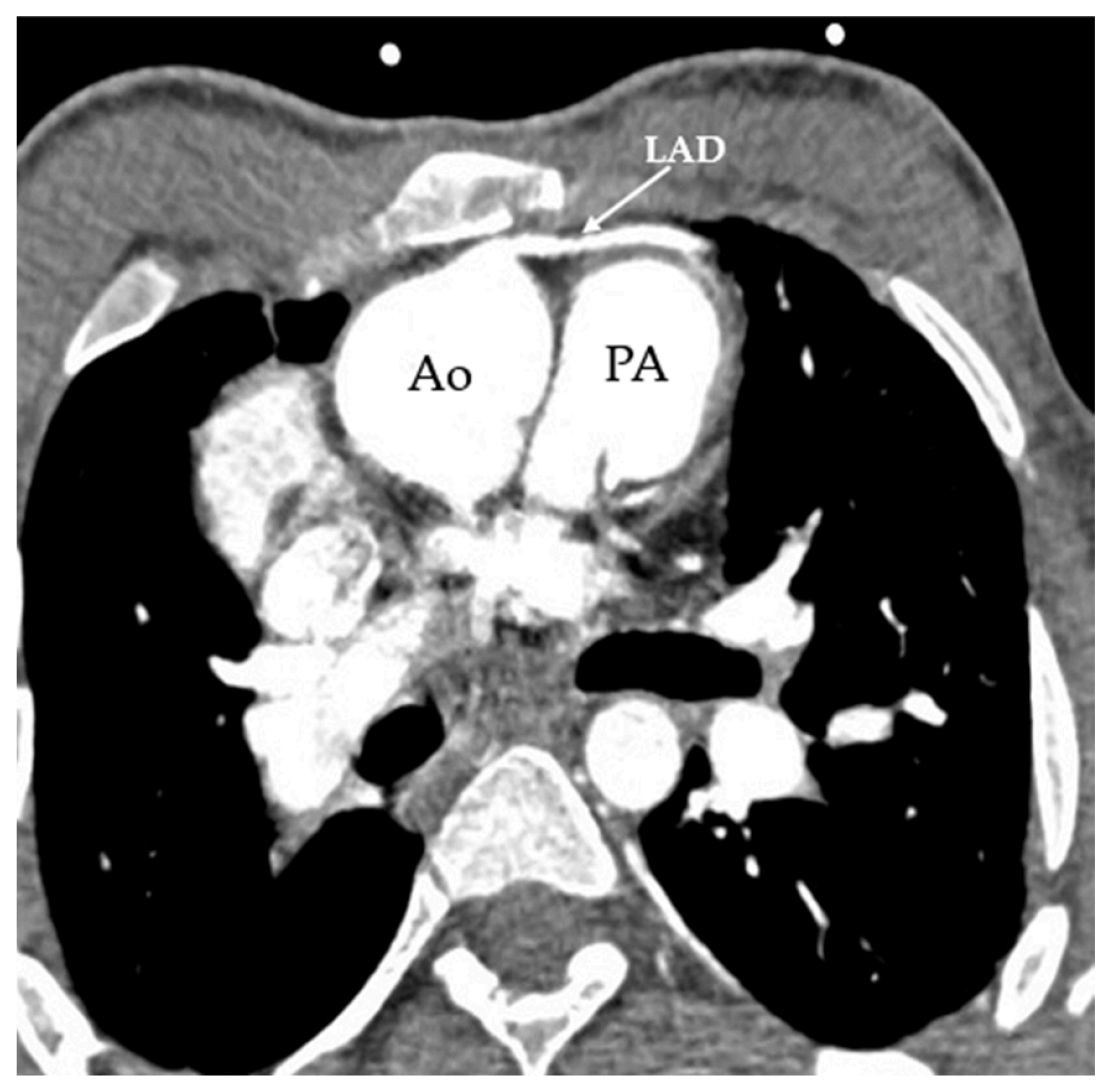
Computed tomography scan showing anomalous left anterior descending (LAD) coronary artery crossing the right ventricular outflow tract in an adult patient with tetralogy of Fallot; Ao: aorta; PA: pulmonary artery; LAD: left anterior descending coronary artery.

Tetralogy of Fallot (TOF) is the most common form of cyanotic CHD, and CA anomalies are present in approximately 4–6% of patients, with some series reporting rates up to 14% [17,19,20]. A meta-analysis encompassing 6956 patients found that 6% had an anomalous CA, of which 72% crossed the RVOT (Figure 5) [19]. The most common anomaly is the LAD artery arising from the right CA and crossing the RVOT, occurring in 3–7% of TOF patients [9,17,19]. Additional anomalies include dual LAD artery, single CA with the left main crossing the RVOT, large conus arteries (6%), and coronary arteriovenous fistulae (4%) [19,20].

The combined risk of encountering an anomalous CA or a large conus artery crossing the RVOT is approximately 10.3% [19]. Pre-operative recognition is critical when planning operations involving the RVOT, including pulmonary valve replacement and conduit placement. The 2018 and 2025 AHA/ACC guidelines recommend that CA anatomy should be delineated before any intervention on the RVOT (Class I recommendation) [6,8,9]. CTA has emerged as the preferred modality for this assessment, with a reported prevalence of coronary anomalies in TOF of 7.6% on CTA [18].

### 3.2. Transposition of the Great Arteries

In dextro-transposition of the great arteries (d-TGA), CA anatomy is highly variable and directly impacts the feasibility and technique of the arterial switch operation (ASO). The most common coronary pattern involves the left CA arising from the left-facing sinus and the right CA from the right-facing sinus, but numerous variants exist, including single CA, intramural CA, and inverted coronary patterns [6,8,21]. Intramural coronary arteries are particularly important, as they represent an independent predictor of both mortality and coronary reintervention after ASO [22].

### 3.3. Congenitally Corrected Transposition of the Great Arteries

In congenitally corrected transposition (ccTGA), the coronary arteries display the anatomy typical of the ventricle they supply, but in a reversed manner. In patients with situs solitus, the right-sided CA supplies the morphologic left ventricle and divides into an anterior descending and circumflex artery, while the left-sided CA encircles the tricuspid valve to supply the morphologic right ventricle [12]. A tendency toward early branching with a short main stem exists, and the coronary arteries may be entrapped in right ventricular fat or muscle. The most common variation is a single CA arising from the right-facing sinus [12].

### 3.4. Double Outlet Right Ventricle

In most patients with double outlet right ventricle (DORV), the coronary arterial orifices are rotated clockwise. With a right-sided and anterior aorta, the coronary pattern resembles that of d-TGA. Reported variations include single CA and origin of the LAD artery from the right CA crossing anteriorly beneath the pulmonary valve across the RVOT [12].

### 3.5. Truncus Arteriosus

Abnormal CA origin and distribution are common in truncus arteriosus and may adversely affect surgical correction. Reported anomalies include single CA origins, abnormally high ostial takeoff above the sinuses of Valsalva, and anomalous coronary courses [12]. Recognition of high takeoff is particularly important when an aortotomy is planned for truncal valve procedures.

### 3.6. Pulmonary Atresia with Intact Ventricular Septum

This lesion is uniquely associated with coronary arterial fistulae and coronary arterial stenoses, which may result in right ventricle–dependent coronary circulation. In severe cases, the coronary circulation is entirely dependent on the hypertensive right ventricle, making decompression of the right ventricle potentially lethal [6,12].

### 3.7. Supravalvar Aortic Stenosis

Coronary artery ectasia is characteristic of supravalvar aortic stenosis (Williams syndrome), resulting from exposure of the coronary arteries to elevated pressures proximal to the supravalvar obstruction [12]. It is also important to notice that the coronary ostia can be involved in supravalvular stenosis and this may require concomitant surgical coronary osteoplasty in addition to relieving supravalvular aortic stenosis. Detection of this abnormality can be particularly difficult even with cardiac catheterization.

### 3.8. Bicuspid Aortic Valve

Patients with bicuspid aortic valve (BAV) have a prevalence of AAOCA that is nearly double that of patients with tricuspid aortic valve [7]. This association should be considered during pre-operative evaluation for aortic valve surgery.

## 4. Postoperative and Acquired Coronary Complications in Repaired ACHD

The introduction of reparative operations involving manipulation, transfer, or reimplantation of the coronary arteries has defined a growing population of patients at risk for acute and chronic coronary complications (Table 4) [8,12,21].

### 4.1. After the Arterial Switch Operation

The ASO is the standard repair for d-TGA, and long-term survival is excellent, with 30-year survival rates of approximately 89–95% [22,23]. However, coronary complications remain a critical concern. Asymptomatic coronary occlusion occurs in 2–7% of ASO patients, and coronary reintervention is required in approximately 2–2.8% [21,22,23]. Freedom from coronary reintervention at 20–25 years is approximately 96% [8,22].

Intimal thickening of the proximal coronary arteries is common after ASO, and obstructive coronary lesions are not rare [6,12]. Compression of the anteriorly positioned proximal coronary arteries by the neopulmonary root after the Lecompte maneuver can also occur [8,12]. Recent studies have identified high-risk coronary features associated with increased risk for ventricular tachycardia and SCD, including intramural course, acute angulation of the left CA, clockwise rotation of the left coronary ostium, and smaller distance between the left coronary ostium and the neopulmonary artery [8]. Intramural CA at the time of ASO is an independent predictor of both mortality (HR 5.2) and coronary reintervention (HR 33.9) [22].

The 2025 ACC/AHA guideline recommends a benchmark assessment of coronary anatomy (via catheter angiography or CTA) in adults after ASO in whom this information has not been obtained, with subsequent investigations largely symptom-driven [6,8]. Emerging data suggest increased risk for acquired CA disease following ASO, and regular assessment of traditional cardiovascular risk factors may be beneficial [8].

A recent multicenter cohort study of 1125 ASO patients with up to 30 years of follow-up confirmed the durability of modern coronary transfer techniques, with coronary complications occurring in 2.8% and reintervention required in 2% [23]. Neoaortic root dilation (z-score ≥ 4) developed in 18%, typically beyond the first decade, representing the dominant late burden alongside right-sided reintervention [23].

### 4.2. After the Ross Procedure

The Ross procedure involves pulmonary autograft replacement of the aortic root, necessitating coronary button transfer. Coronary button aneurysm is a recognized complication, arising from the unsupported areas of the pulmonary autograft exposed to systemic arterial pressure [12]. Preventive measures include suturing the autograft onto the native aortic annulus, reinforcing the proximal suture line with fabric, avoiding redundant right ventricular muscle, and considering encasement of the autograft in a Dacron tube [12].

**Figure 6:**
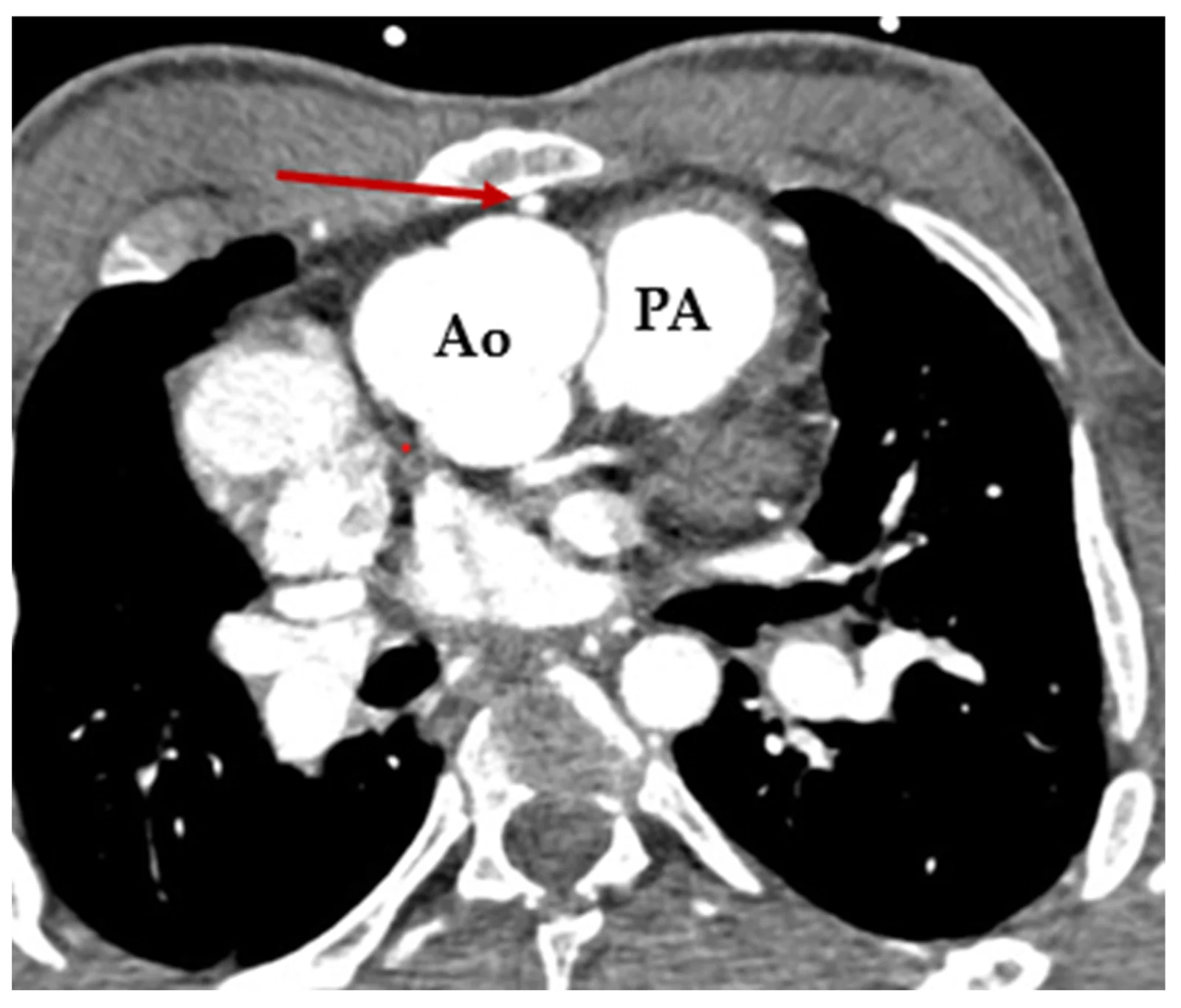
Computed tomography scan showing the dilated aortic root with the more cephalad and anteriorly displaced right coronary artery button (red arrow) in a patient who underwent Ross procedure. This can constitute a challenge during reoperation; Ao: aorta; PA: pulmonary artery.

Progressive autograft dilation may lead to cephalad and anterior displacement of the coronary buttons (Figure 6), potentially causing coronary kinking or compression. Long-term surveillance with cross-sectional imaging is essential.

### 4.3. After ALCAPA Repair

Adults who underwent ALCAPA repair in childhood may have residual ischemia, graft stenosis, or patchy myocardial fibrosis from pre-operative ischemia [9]. Supra-pulmonary arterial stenosis, baffle leaks, and baffle stenosis have been reported after Takeuchi baffle repair. Late aortic regurgitation and residual mitral valve disease are additional concerns [9]. Coronary flow reserve abnormalities and proximal, mid-vessel, and distal coronary obstructions have been noted and treated with balloon dilation, stenting, and reoperation [9].

### 4.4. Iatrogenic Coronary Injury During Congenital Heart Surgery

CA injury may occur during redo sternotomy, RVOT reconstruction, conduit placement, or aortotomy in patients with unrecognized coronary anomalies. Pre-operative CT imaging to define the relationship of coronary arteries to the sternum and planned surgical field is strongly recommended [5].

### 4.5. Accelerated Atherosclerosis in the ACHD Population

As the ACHD population ages, the interaction between congenital coronary anomalies and acquired atherosclerotic disease becomes increasingly relevant. The long-term coronary fate of the ASO generation, now entering their late 30s and 40s, remains unknown, particularly regarding the impact of traditional cardiovascular risk factors on surgically manipulated coronary arteries [6,8,21].

## 5. Multimodality Imaging for Coronary Assessment in ACHD

The evaluation of CA anomalies in ACHD requires a multimodality imaging approach, with each modality offering complementary strengths and limitations [5,10,17,24].

### 5.1. Echocardiography

Transthoracic echocardiography (TTE) is the first-line screening tool for coronary anomalies, particularly in younger patients, and can identify the origin and proximal course of the coronary arteries in many cases [17]. The 2020 ASE multimodality assessment guidelines provide detailed recommendations for echocardiographic evaluation of congenital coronary anomalies [17]. However, sensitivity decreases in older adults due to limited acoustic windows, and the right CA is generally harder to image than the left [5]. TTE is particularly valuable for establishing baseline functional assessment and identifying regional wall motion abnormalities suggestive of ischemia.

### 5.2. Coronary Computed Tomography Angiography

Coronary CTA has become the gold standard for anatomic assessment of anomalous coronary origin and course in adults [5,7,10]. CTA provides submillimeter spatial resolution for delineation of coronary anatomy in relation to the great arteries and myocardial geometry, allowing assessment of origin, course, luminal narrowing, intramural segment length, ostial morphology, and concomitant atherosclerosis [7]. The reported prevalence of coronary anomalies is higher with CTA than with invasive angiography (7.9% vs. 2.1%) [10].

The 2025 ACR Appropriateness Criteria recommend contrast-enhanced CTA of the coronary arteries as the initial imaging modality for suspected CA anomalies in adults (rated “usually appropriate”) and as the recommended test for presurgical planning [10]. High-risk anatomic features identifiable on CTA include slit-like or fish-mouth-shaped orifice, acute angle takeoff (<45°), intramural course and its length, inter-arterial course, and proximal vessel hypoplasia [6,7].

CTA also plays a critical role in pre-operative planning for redo sternotomy in ACHD patients, defining the relationship of coronary arteries, conduits, and vascular structures to the sternum [5]. In repaired TOF patients being considered for pulmonary valve replacement, CTA is recommended to delineate coronary anatomy before any RVOT intervention [9,17,18].

### 5.3. Cardiac Magnetic Resonance Imaging

Cardiac MRI (CMR) offers several unique advantages for coronary assessment in ACHD, including the absence of ionizing radiation, unrestricted imaging planes, and the ability to assess ventricular function, myocardial perfusion, and fibrosis in a single examination [5,6,24,25]. Whole-heart coronary MR angiography can identify coronary origins with a success rate of approximately 94% [25]. CMR is particularly valuable for:

- Identifying myocardial fibrosis via late gadolinium enhancement (LGE), which may serve as a substrate for ventricular arrhythmias and provides additional risk stratification [24,25,26].
- Stress perfusion imaging (dobutamine or regadenoson) to assess for inducible ischemia [8,17,25].
- Serial follow-up without cumulative radiation exposure, particularly important in the young ACHD population [6,25].
- Assessment of ventricular function and valvular pathology in the same examination [5,6].

The 2025 ACR Appropriateness Criteria rate both contrast-enhanced and non-contrast coronary MRA as “usually appropriate” alternatives to CTA for initial evaluation of suspected coronary anomalies [10]. However, CTA generally provides higher diagnostic consistency across centers and more accurate depiction of distal coronary segments [24].

### 5.4. Invasive Coronary Angiography and Role of Invasive Physiologic Assessment

Invasive coronary angiography remains important when there is concern about coronary stenosis, when concomitant hemodynamic evaluation is needed, or when intravascular ultrasound (IVUS) and fractional flow reserve (FFR) assessment are required [5,6,8]. In patients with AAOCA and high-risk anatomic features, IVUS and FFR may provide additional diagnostic information regarding the functional significance of proximal narrowing [8]. However, invasive angiography has limitations in defining the three-dimensional relationship of anomalous vessels to surrounding structures and has been largely supplanted by CTA for anatomic assessment [7,10].

Invasive physiological assessment techniques such as fractional flow reserve (FFR), instantaneous wave-free ratio (iFR), and intracoronary Doppler flow velocity measurements are emerging modalities to assess the hemodynamic significance of coronary artery anomalies in adults with congenital heart disease.

These tools allow direct measurement of coronary flow impairment caused by anatomical anomalies or surgical reconstructions and can aid in distinguishing lesions that warrant intervention from those safely observed. For example, in AAOCA patients or post-arterial switch operation patients with borderline imaging findings, physiological assessment can identify ischemia-inducing stenoses.

While data remain limited, early studies suggest that combining physiological measurements with imaging enhances diagnostic accuracy and guides tailored therapies. Technical challenges include catheter access and interpretation in anomalous anatomy, requiring experienced operators.

Routine application of these techniques in ACHD care is expected to expand, warranting further clinical trials to validate prognostic utility and impact on outcomes.

### 5.5. Stress Imaging and Functional Assessment

Physiologic assessment of ischemia is a critical component of risk stratification in AAOCA. The 2025 ACC/AHA guideline recommends exercise or dobutamine stress testing with echocardiographic, nuclear, or MR imaging over vasodilator stress testing, as exercise and dobutamine better reflect the dynamic conditions that provoke ischemia in AAOCA [8]. Regadenoson stress CMR perfusion has shown correlation with catheterization data in ASO patients, with approximately one-third of perfusion studies in symptomatic patients identifying a perfusion defect [8].

However, the sensitivity of stress testing in AAOCA is limited, and a normal stress test does not exclude SCD risk; some patients have experienced SCD despite normal stress electrocardiograms [8]. This limitation must be communicated during shared decision-making.

### 5.6. Comparison of Imaging Modalities

Each imaging modality offers distinct advantages and limitations in the ACHD population (Table 5). CTA provides the highest spatial resolution for anatomic assessment but involves ionizing radiation. CMR avoids radiation and provides functional and tissue characterization data but has lower spatial resolution for coronary imaging and longer acquisition times. Echocardiography is widely available and radiation-free but has limited sensitivity in adults. Invasive angiography provides hemodynamic data and interventional capability but is invasive and limited in three-dimensional anatomic assessment [5,10,24].

## 6. Risk Stratification

Risk stratification of coronary anomalies in ACHD integrates anatomic features, physiologic assessment, patient age, and clinical context (Table 6) [5,6,7,8].

### 6.1. Anatomic Risk Factors

The 2018 and 2025 AHA/ACC guidelines identify several anatomic features that may relate to the clinical importance of AAOCA and risk of SCD: [6,8].

- Slit-like or fish-mouth-shaped orifice, more commonly seen in anomalous right CA from the left sinus.
- Acute angle takeoff, particularly with high takeoff.
- Intra-mural course; the presence and length of the intramural segment correlate with preoperative symptoms; longer segments are associated with worse outcomes [5,7,22].
- Interarterial course, passage between the aorta and pulmonary artery
- Proximal vessel hypoplasia
- Dominance of the anomalous vessel; a dominant anomalous vessel carries higher risk

The pathophysiology of ischemia in AAOCA involves multiple mechanisms, including flap-like closure of the slit-like orifice, acute angle takeoff with kinking, compression of the intramural segment by the aortic commissure, compression between the great vessels accentuated by exercise-related expansion of the pulmonary artery, and coronary spasm from endothelial injury [7,12].

### 6.2. Physiologic Risk Factors

Inducible ischemia on stress testing, reduced FFR, and regional wall motion abnormalities support the clinical significance of an anatomic anomaly [8]. However, the absence of ischemia on stress testing is not completely reassuring, as SCD has occurred in patients with normal stress evaluations [8].

### 6.3. Age-Related Considerations

AAOCA is more commonly invoked as the cause of SCD in patients younger than 35 years, in whom atherosclerotic coronary disease is less prevalent [6]. However, death has been attributed to AAOCA in patients of all ages, and there does not appear to be an age beyond which the anomaly is no longer relevant [6]. In older ACHD patients, the interaction between congenital coronary anomalies and acquired atherosclerosis adds complexity to risk assessment [5].

### 6.4. The Challenges in the Asymptomatic Patient

Management of the asymptomatic patient with AAOCA remains one of the most controversial areas in the field. The 2025 ACC/AHA guideline supports surgical repair in asymptomatic patients with high-risk features, particularly in asymptomatic middle-aged patients with features such as inter-arterial course, intramural segments, slit-like ostia, or evidence of ischemia on functional testing. For truly asymptomatic individuals without high-risk morphology and absent ischemia, conservative management with close follow-up may be appropriate.

Risk stratification incorporating multimodality imaging—including coronary CT angiography to define anatomy—and physiological assessments helps guide decision-making. Individualized discussion addressing patient activity level, comorbidities, and preferences is essential, as the balance between surgical risks and sudden death prevention remains nuanced.

**Figure 7:**
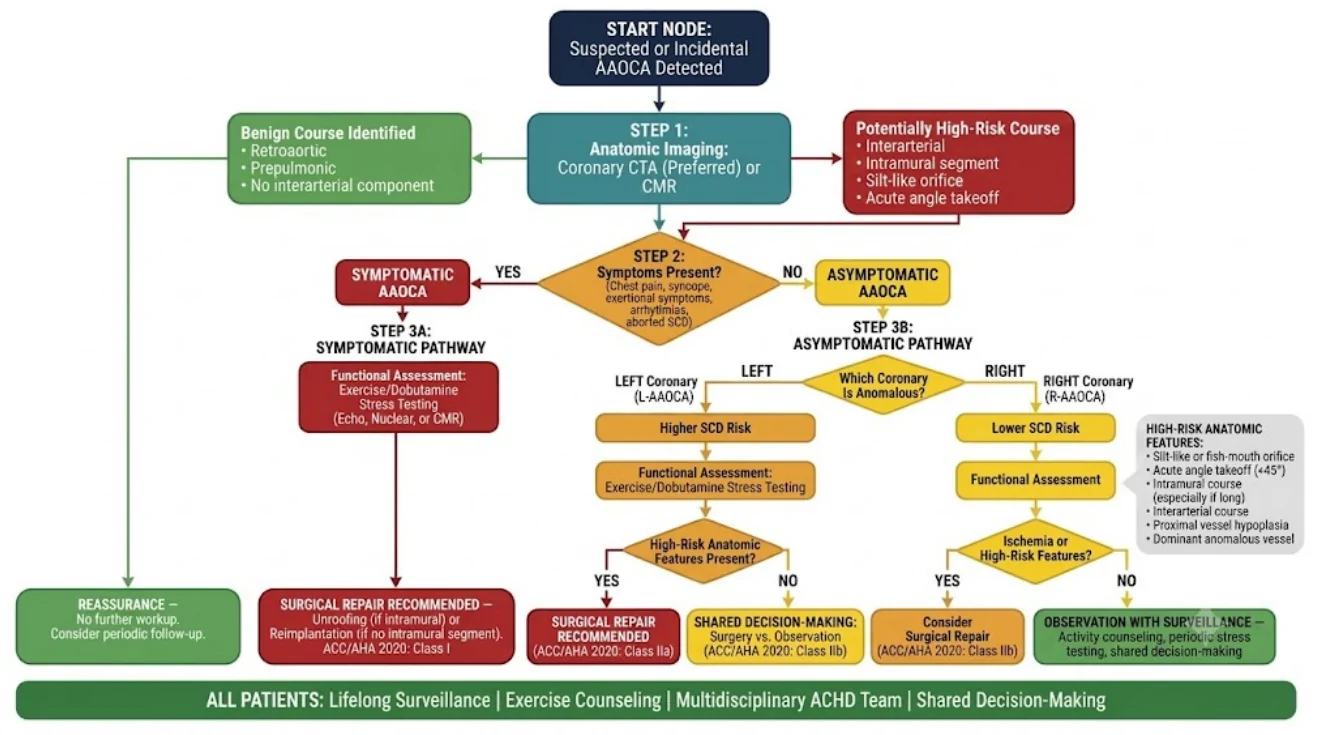
Risk Stratification and Management Algorithm. Google Gemini 2.5 Pro was used as a graphical/design aid to produce the visual representation of an author-defined concept; it was not used to generate the underlying scientific concept, data, findings, or medical information [27].

More prospective data are needed to clarify optimal strategies, but multidisciplinary evaluation in centers with ACHD and advanced imaging expertise is currently advocated. A clinical algorithm integrating these considerations is presented in (Figure 7) [8].

## 7. Management Strategies

### 7.1. Conservative Management and Activity Restriction

Conservative management, including medical therapy and/or activity restriction, can be appropriate for adults with coronary arterial anomalies who exhibit low-risk anatomical features and no evidence of inducible ischemia. Such an approach should be guided by careful shared decision-making between the patient and clinician. While beta-blockers may be considered to alleviate symptoms, there is currently no randomized trial evidence supporting their routine use in patients with anomalous aortic origin of a coronary artery (AAOCA). Recommendations regarding exercise restriction should be individualized, taking into account the specific type of coronary anomaly, associated anatomical risk factors, and functional testing results [8].

### 7.2. Surgical Management

Multiple surgical and interventional techniques are available for the management of coronary anomalies, with selection guided by the specific anomaly type and anatomic features.

#### 7.2.1. Anomalous Aortic Origin of a Coronary Artery

**Figure 8:**
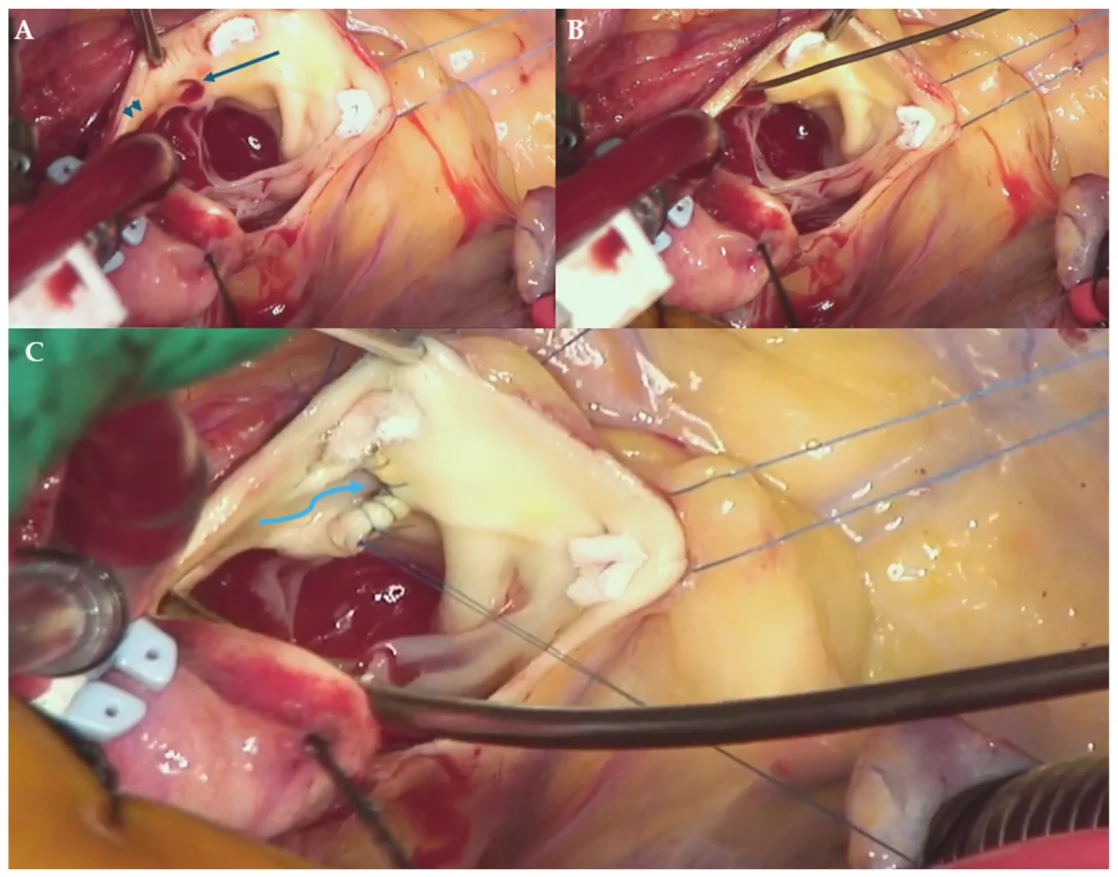
(**A**–**C**): Intraoperative photos demonstrating the technique of unroofing of anomalous origin of the right coronary artery from the left coronary sinus of Valsalva of the aortic root. (**A**) the ostium of the right coronary artery (green arrow) is seen to the left of the commissure between the right and left coronary sinuses and in close proximity to the left coronary artery ostium (multiple arrow heads), (**B**) a probe is passing through the right coronary artery ostium to confirm the intramural course, (**C**) the intramural course of the right coronary artery is unroofed, thus translocating the ostium back to the right coronary sinus (blue arrow).

**Figure 9:**
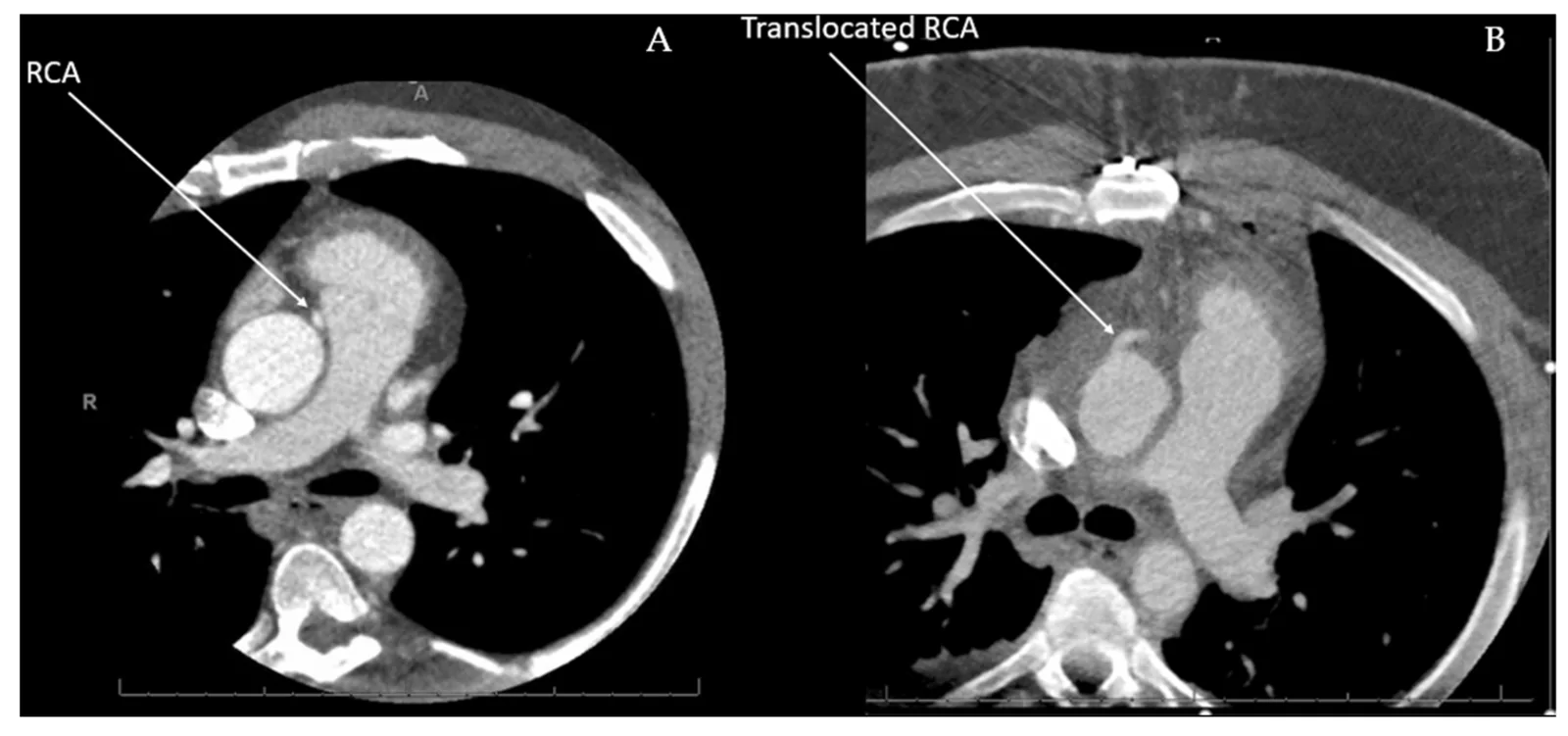
(**A**,**B**): If the anomalous coronary artery does not have an intramural segment, then translocation of the entire ostium can provide an alternate solution. (**A**) preoperative and postoperative (**B**) computed tomography scan images showing the inter-arterial course of the right coronary artery (RCA) with no intramural segment and in (**B**) after being translocated to the ascending aorta; RCA: right coronary artery.

**Figure 10:**
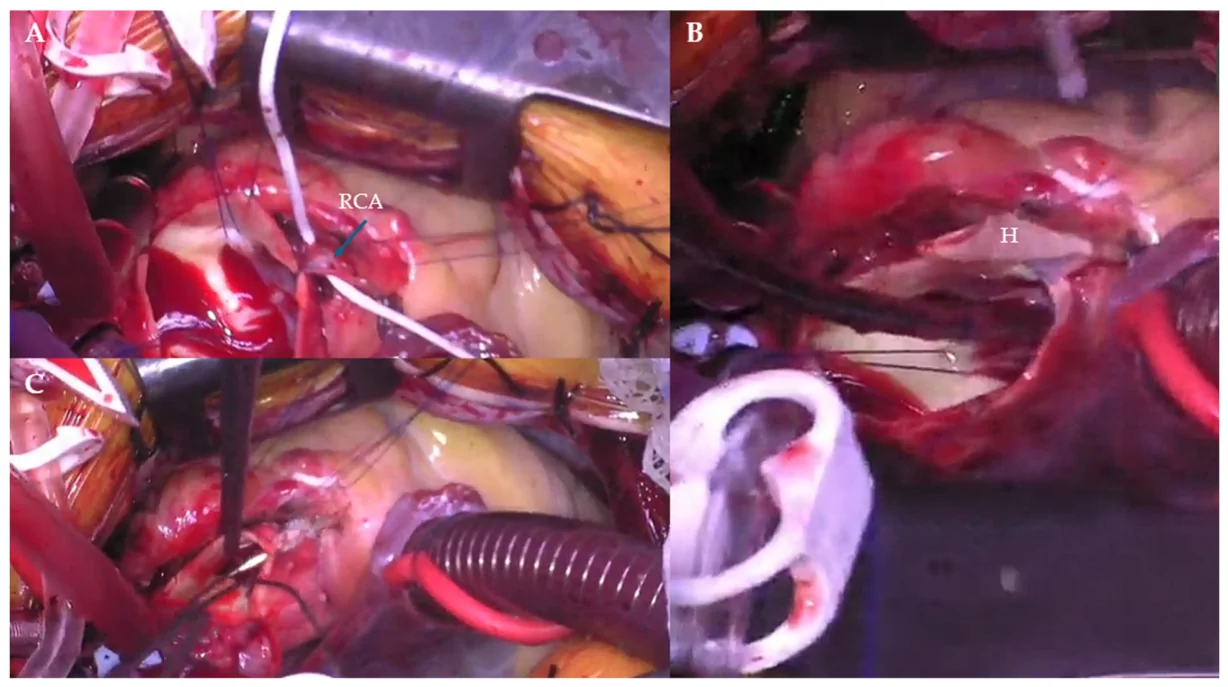
(**A**–**C**): Intraoperative photos showing creation of a neo-ostium which represents an alternative solution for anomalous coronary artery origin from the wrong sinus of Valsalva of the aortic root and it is especially useful when the abnormal ostium is located close to the commissure of the aortic valve. (**A**) the right coronary artery (RCA) is dissected as it crosses close to the aortic wall and an arteriotomy is created followed by a side-to-side anastomosis between the right coronary arteriotomy and the corresponding location in the right coronary sinus of the aortic root, (**B**) the roof of the anastomosis can be augmented with fresh autologous pericardial or homograft (H) patch, (**C**) the final appearance of the neo-ostium with a probe passing through it to confirm its adequate size, notice the original ostium of the right coronary artery is left intact; RCA: right coronary artery; H: homograft patch.

Surgical approaches for AAOCA include [7,8,28]:

- Unroofing with or without osteoplasty (Figure 8A–C): The most commonly used technique for AAOCA with an intramural component. The intramural segment is incised and marsupialized, creating a neo-ostium in the correct sinus. The incision typically extends 5–15 mm. Results are excellent with no reported operative mortality in large series. Potential complications include aortic dissection, aortic regurgitation from commissure prolapse, and persistent symptoms due to recurrent stenosis from incomplete unroofing [7,28].

- Coronary reimplantation: Preferred when the intramural segment is short, when unroofing would place the ostium too close to the intercoronary pillar, or when there is no intramural segment with an inter-arterial course (Figure 9A,B). A recent series of 230 patients reported no early or late deaths, with a 2.6% reoperation rate; notably, no reoperations were required in the reimplantation group compared to 5.8% in the unroofing cohort [7,28].

- Neo-ostium creation (Figure 10A–C): An alternative for patients with an intramural course, involving passage of a probe through the intramural segment and creation of a new ostium in the correct sinus [12].

- Pulmonary artery translocation: Used when the anomalous course is interarterial but not intramural, and the ostia are close together. The pulmonary artery is moved anteriorly and leftward to relieve compression [12].
- Coronary artery bypass grafting (CABG): Reserved for older adults with concomitant atherosclerotic disease or when anatomic correction is suboptimal. Concern about long-term graft patency due to competitive flow makes CABG less desirable in young patients [7,8,14].

The selection of surgical technique is guided by the presence, length, and location of the intramural segment (Figure 4). In a protocolized single-institution experience of 148 consecutive patients undergoing surgical unroofing (median age 44.4 years; 130 with anomalous RCA, 19 with anomalous LCA), there were 2 early deaths (1%), both in reoperative patients. Over a median follow-up of 9.5 years, late survival at 10 and 15 years was 94.5%, and no patients had evidence of ischemia related to the unroofing [29]. In the largest contemporary series of 230 patients undergoing AAOCA repair (median age 17 years), no early or late deaths occurred with a median follow-up of 4 years. Reoperations were required in 2.6%, with no reoperations in the reimplantation group compared to 5.8% in the unroofing cohort, leading the authors to favor coronary reimplantation as their current preferred technique [28].

A recent large Italian cohort study of 173 adults with AAOCA identified by CTA over 20 years found that only 6% underwent surgical repair, with the majority managed conservatively. After a median 37-month follow-up, mortality and major adverse cardiac events were similar between AAOCA patients and matched controls with normal coronary anatomy. Notably, only concomitant obstructive atherosclerotic disease, not AAOCA subtype or ischemia, was associated with a higher event rate, underscoring the importance of atherosclerotic risk factor management in this population [30].

#### 7.2.2. Anomalous Origin of a Coronary Artery from the Pulmonary Artery

Surgical repair of ALCAPA in adults aims to establish a two-coronary system perfused by the left heart. Direct coronary arterial translocation to the aorta is the preferred technique, offering the advantage of avoiding late graft stenosis [12]. When mobilization of a sufficient cuff of pulmonary artery to reach the aorta is not feasible, alternatives include a short interposition graft (e.g., polytetrafluoroethylene), construction of an intrapulmonary baffle (Takeuchi procedure), or bypass grafting [12]. A systematic review of 279 adult ALCAPA cases found that the mean age at surgery was 37.5 years, and surgical repair was associated with improvement in left ventricular function, decrease in ventricular dilation, relief of angina, and protection of the mitral apparatus [15,16].

#### 7.2.3. Coronary Artery Fistulae

Management of coronary artery fistulae requires individualized assessment by a multidisciplinary team. The 2018 AHA/ACC guideline recommends closure for symptomatic fistulae and for large fistulae with evidence of right ventricular enlargement from a significant left-to-right shunt [6]. Postoperative myocardial infarction occurred in 11% of a surgical series of 46 patients, attributed to low flow in the dilated coronary artery proximal to fistula closure [6,31].

Transcatheter closure has become the first-line approach for selected medium to large or symptomatic coronary artery fistulae. A comprehensive review from the Mayo Clinic reported a 90% acute procedural success rate in 45 patients with 56 fistula closures, using coils, Amplatzer vascular plugs, and covered stents [32]. Complications included device migration (3 patients), myocardial infarction (4 patients, 18%), and intracranial hemorrhage (1 patient) [31]. Recanalization remains a concern, with rates of 4 of 27 fistulae (15%) at a median of 423 days in one angiographic series [32]. Follow-up coronary imaging with CTA or angiography at 1 to 5 years is recommended to evaluate for recanalization [32].

Surgical options include epicardial ligation or division (best suited for terminal fistulae), trans-arterial closure with aneurysmorrhaphy, transcardiac chamber closure, tangential arterioplasty, and coronary artery bypass grafting when distal coronary flow is compromised [12]. Postoperative anticoagulation with warfarin for 3–6 months is recommended because of low flow in the dilated segment of the native coronary artery [12]. Patients with large coronary aneurysms are best treated with concomitant surgical bypass and oral anticoagulation to reduce perioperative myocardial infarction risk [32,33].

#### 7.2.4. Post-Arterial Switch

Coronary complications after the arterial switch operation may include ostial stenosis or distortion, proximal kinking due to neoaortic root dilation, and extrinsic compression by the neopulmonary root. Preoperative coronary CTA is essential to define the mechanism of obstruction and plan safe reentry. Surgical options include:

- Coronary ostial patch angioplasty: For focal ostial stenosis at the reimplantation site.
- Coronary reimplantation revision: For malposition or kinking, which may be combined with neoaortic root replacement when significant root dilation is present.
- Coronary artery bypass grafting (CABG): When direct repair is not feasible; arterial conduits are preferred in younger patients.
- Percutaneous coronary intervention (PCI): May be considered for selected focal lesions, though long-term data remain limited.

The choice of strategy should be individualized through multidisciplinary discussion.

#### 7.2.5. Post-Ross Procedure

Late coronary complications after the Ross procedure arise primarily from progressive autograft dilation, which displaces the coronary buttons and may cause ostial kinking or angulation. Coronary button aneurysms may develop where unsupported autograft tissue is exposed to systemic pressure. Surgical options include:

- Coronary ostial revision or patch repair: For focal ostial stenosis or distortion.
- Coronary button aneurysm excision: With reconstruction of the coronary ostium.
- Neo-aortic root replacement with coronary reimplantation: When significant autograft dilation is the primary mechanism of coronary compromise.
- Root reinforcement or wrapping: To stabilize root dimensions in patients with moderate dilation and preserved valve function before coronary compromise develops.
- CABG: When direct coronary repair is not feasible.

Long-term surveillance with cross-sectional imaging is essential to monitor autograft dimensions and coronary button integrity.

#### 7.2.6. Myocardial Bridging

**Figure 11:**
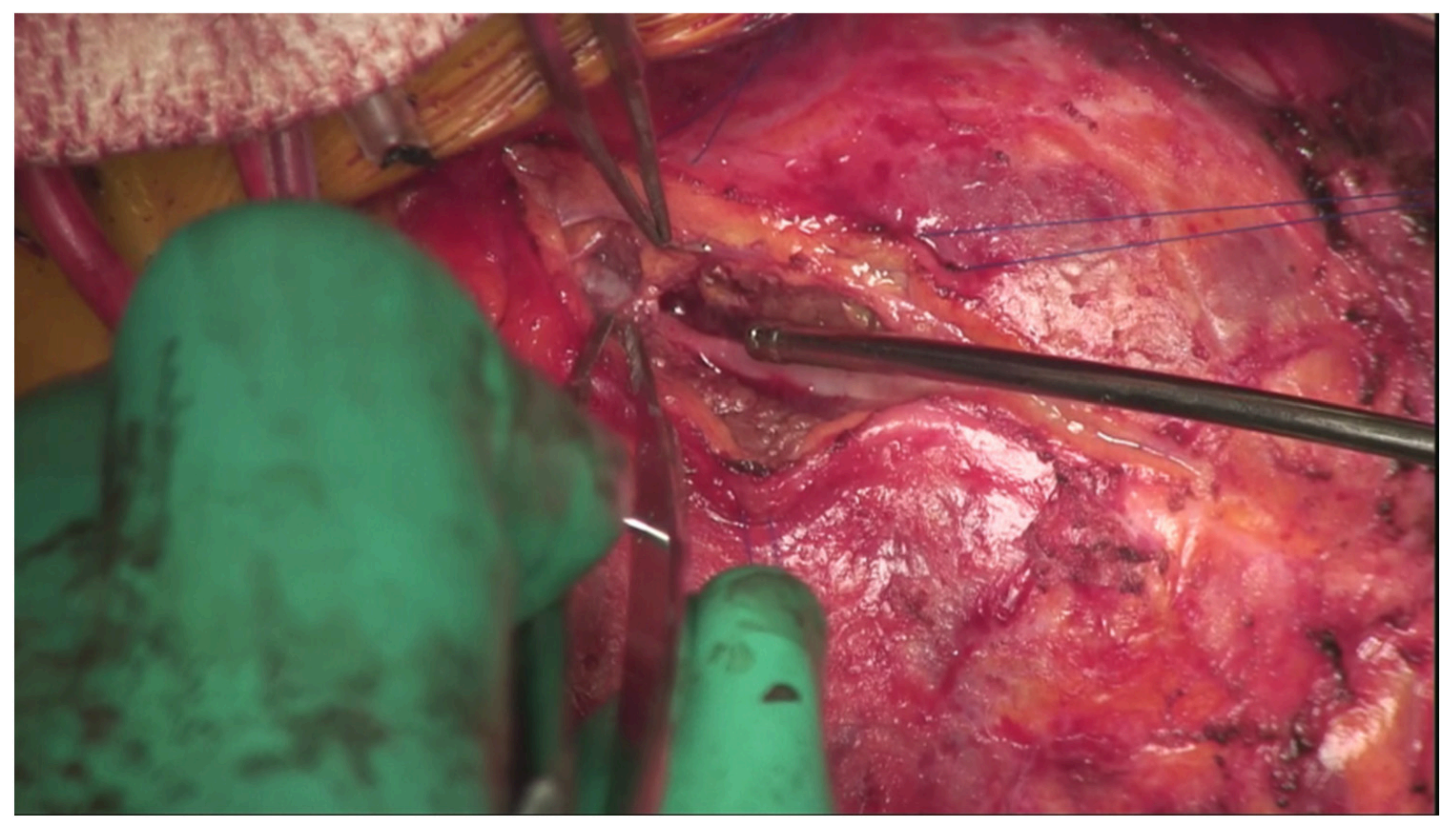
Intraoperative photo showing completely unroofed left anterior descending coronary artery. Notice the long and deep myocardial bridge that was covering the artery.

Surgical intervention is reserved for patients with refractory symptoms despite optimal medical therapy and objective evidence of ischemia. Preoperative coronary CTA is valuable for defining the depth, length, and anatomic relationships of the bridge. Surgical options include:

- Surgical myotomy (unroofing): The primary treatment, involving division of overlying myocardial fibers to release the tunneled artery. Complete unroofing is important to prevent residual compression, with care to avoid injury to septal perforators or the right ventricular cavity in deep bridges (Figure 11).

- CABG: Considered when unroofing carries unacceptable risk due to a very deep intramyocardial course or when concomitant coronary artery disease is present. Long-term graft patency may be compromised by competitive flow through the native artery.
- Coronary stenting: Generally, not recommended due to high rates of in-stent restenosis and stent fracture from repetitive systolic compression.

### 7.3. AHA/ACC Versus ESC Guideline Comparison

Important divergences exist between the AHA/ACC and ESC guidelines regarding the management of coronary anomalies in ACHD, as reflected in the management algorithm (Figure 3) and detailed in (Table 7). The 2018 AHA/ACC guideline provides a Class IIa recommendation for surgery in asymptomatic patients with anomalous left CA from the right sinus, even in the absence of symptoms or ischemia, reflecting the high SCD risk associated with this anatomy [6]. The 2020 ESC guideline takes a more conservative approach, recommending surgery primarily when ischemia is documented and giving a Class III recommendation against surgery in asymptomatic patients without high-risk features [7]. The 2025 ACC/AHA guideline maintains support for surgical repair in asymptomatic patients with high-risk features but acknowledges that watchful waiting with shared decision-making may also be appropriate, particularly in asymptomatic middle-aged patients without high-risk features [8].

For anomalous right CA from the left sinus, both guidelines agree that management of asymptomatic patients is controversial. The AHA/ACC guideline provides a Class IIb recommendation for either surgery or continued observation, while the ESC guideline recommends against surgery in the absence of documented ischemia [7].

### 7.4. Exercise and Sports Participation

The 2025 AHA/ACC Scientific Statement on competitive sports participation provides detailed recommendations for athletes with coronary anomalies [34]. Key recommendations include:

- Inter-arterial left AAOCA: Surgical intervention should be considered regardless of symptoms or ischemia assessment results. Competitive athletes should not participate in competitive sports if left unrepaired [34].
- Inter-arterial right AAOCA: Competitive sports participation is reasonable if there are no exertional symptoms or inducible ischemia, with shared decision-making and longitudinal surveillance. Surgical intervention should be considered if there is evidence of ischemia or concerning symptoms [34].
- ALCAPA: Competitive athletes should not participate in competitive sports (except low-intensity class IA sports) until surgical repair, which is indicated regardless of symptoms, ischemia evaluation results, LV function, or presence of ischemic mitral regurgitation [34,35].
- Myocardial bridging: Asymptomatic athletes with incidental myocardial bridging can participate in competitive sports. Symptomatic athletes should undergo ischemia assessment, and those with persistent ischemia after treatment should be restricted from competitive sports [34].
- After surgical repair: Resumption of competitive sports can proceed after complete sternal healing and testing showing no evidence of myocardial ischemia and no complex ventricular arrhythmias [34].

## 8. Long-Term Outcomes and Surveillance

### 8.1. Outcomes After Surgical Repair of AAOCA

Surgical repair of AAOCA can be performed with extremely low mortality and a low incidence of reoperation. In the largest contemporary series, no early or late deaths occurred among 230 patients, with a 2.6% reoperation rate at a median follow-up of 4 years [28]. A protocolized unroofing approach in 148 patients demonstrated 94.5% survival at 10 and 15 years, with no evidence of ischemia related to the unroofing in any patient during follow-up [29]. A comprehensive review of published data identified 20 studies with at least 20 AAOCA patients and more than 1 year of follow-up, finding that AAOCA-related death is rare in available follow-up, and surgical repair appears safe and effective in relieving symptoms in up to 97% of patients [36].

However, important caveats exist. No randomized trials have compared surgical repair with conservative management. In conservatively managed cohorts, 27% of patients reported an increase in chest pain or diminished exercise tolerance during follow-up [36]. The long-term rates of patency of neo-ostia after unroofing remain incompletely characterized, and subclinical evidence of postoperative myocardial ischemia has been found in some patients despite patent neo-ostia and absence of symptoms [12].

### 8.2. Long-Term Coronary Outcomes After the Arterial Switch Operation

The ASO generation represents a unique and growing population requiring lifelong coronary surveillance. Freedom from coronary events was 88.1% at 22 years in one long-term study [21]. The 2025 ACC/AHA guideline recommends a benchmark assessment of coronary anatomy in adults after ASO, in whom this information has not been obtained, with subsequent investigations largely symptom-driven [8]. No existing data support routine serial coronary-specific imaging after the coronary anatomy is defined, although such imaging may be reasonable if high-risk features are identified [8].

Detection of subclinical myocardial ischemia after ASO remains challenging. Clinical evaluation, ECG, and echocardiography possess low sensitivity for detecting coronary insufficiency [21]. Exercise-induced myocardial perfusion defects are present by SPECT in 5–10% of patients, whereas symptoms and ST-segment depression are rare and of unclear significance [21]. Regadenoson stress CMR perfusion has shown promise, with approximately one-third of perfusion studies in symptomatic patients identifying a perfusion defect [8].

Emerging data suggest increased risk for acquired coronary artery disease following ASO, and it may be beneficial to ensure regular assessment and treatment of traditional cardiovascular risk factors [8]. Given that the oldest ASO patients are now in young adulthood, natural history studies have not yet determined whether there is increased risk for atherosclerotic coronary artery disease after ASO [6,8].

### 8.3. Surveillance Protocols and Imaging Intervals

The 2020 ACC/AHA/ASE/HRS/ISACHD/SCAI/SCCT/SCMR/SOPE Appropriate Use Criteria for Multimodality Imaging guide imaging intervals for follow-up of patients with congenital heart disease [27]. For patients after ASO, echocardiography is recommended annually, with CMR every 3–5 years for stable patients and every 1–2 years for those with neoaortic root dilation or significant branch pulmonary artery stenosis [8]. For patients with AAOCA, surveillance should include periodic stress testing and cross-sectional imaging, with intervals individualized based on the specific anomaly, surgical repair status, and risk features [8].

### 8.4. Pregnancy Considerations

Women with coronary anomalies require pre-conception counseling and multidisciplinary management during pregnancy. The hemodynamic changes of pregnancy, including increased cardiac output, heart rate, and blood volume, may exacerbate ischemia in patients with hemodynamically significant coronary anomalies. Women with unrepaired ALCAPA or symptomatic AAOCA should be counseled regarding the risks of pregnancy and considered for surgical repair before conception. After successful surgical repair with no residual ischemia, pregnancy is generally well-tolerated, although close surveillance is recommended.

## 9. Knowledge Gaps, Controversies, and Future Directions

### 9.1. Lack of Prospective Data on Natural History

No prospective studies have defined the natural history of AAOCA in adults, and no randomized trials have compared surgical repair with conservative management. The low event rate in AAOCA makes prospective outcome studies challenging, and multicenter registries are needed to refine risk stratification [30]. The recent Italian cohort study demonstrating that concomitant obstructive atherosclerotic disease, rather than AAOCA subtype or ischemia, was the primary determinant of adverse events in adults highlights the complexity of risk assessment in this population [30].

### 9.2. The Asymptomatic Anomalous Right Coronary Artery Controversy

Management of asymptomatic patients with an anomalous right coronary artery from the left sinus remains one of the most debated topics in the field. The AHA/ACC and ESC guidelines diverge on this issue, and no prospective data exist to resolve the controversy [7,8]. The 2025 ACC/AHA guideline acknowledges that in asymptomatic middle-aged patients without high-risk features, surgery has no documented benefit on symptoms or long-term survival [8].

### 9.3. The Arterial Switch Generation: Unknown Long-Term Coronary Fate

The first large cohorts of ASO patients are now entering their late 30s and 40s, and the interaction between surgically manipulated coronary arteries and traditional cardiovascular risk factors remains unknown [8,21]. Whether these patients will develop accelerated atherosclerosis and whether standard preventive strategies are sufficient are critical unanswered questions. A recent multicenter cohort study with 30-year follow-up confirmed the durability of modern coronary transfer techniques but identified neoaortic root dilation as the dominant late burden [23].

### 9.4. CT-Derived Fractional Flow Reserve

CT-FFR is an emerging noninvasive technique that uses computational fluid dynamics to model coronary flow, pressure, and resistance from standard CTA data. CT-FFR may have utility in patients with AAOCA, coronary artery stenosis after reimplantation as part of CHD surgery, and those with Kawasaki disease or cardiac transplantation [37]. However, a retrospective study of CT-FFR in adults with AAOCA did not show a difference in CT-FFR values between those considered at higher risk based on anatomic features and those at lower risk in a 5-year follow-up [37]. Prospective CT-FFR in AAOCA patients has been described only in case reports, and the low rate of adverse events would hinder investigations of its value for risk stratification [37]. An international expert consensus on CT-FFR has explored its application in non-atherosclerotic diseases, including myocardial bridging and AAOCA, but validation in these populations remains limited [38].

### 9.5. Photon-Counting CT

Photon-counting CT (PCCT) represents a transformative advancement in cardiac imaging, offering superior spatial resolution, enhanced tissue characterization, multienergy capabilities, and reduced radiation dose compared with conventional CT [39,40]. PCCT enables superior coronary lumen and plaque evaluation, even in complex cases with severe calcification or smaller coronary stents, and offers improved visualization of cardiac anatomy and myocardial tissue characterization [39,40,41]. In the ACHD population, PCCT may provide more precise delineation of anomalous coronary anatomy, intramural segments, and ostial morphology, potentially reducing the need for invasive angiography. However, challenges, including high costs, increased data demands, and a need for standardized protocols, remain [40].

### 9.6. Four-Dimensional Flow MRI

Four-dimensional flow MRI provides velocity information in all three orthogonal planes over the entire cardiac cycle, enabling comprehensive hemodynamic assessment, including vorticity, helicity, wall shear stress, kinetic energy loss, and pressure gradients [38,42]. In complex CHD, 4D flow excels at depicting streaming and multilevel shunt quantification [37]. While 4D flow has been increasingly used in aortopathies and complex CHD, its specific application to coronary anomaly assessment remains investigational. Potential applications include assessment of coronary flow dynamics in patients with AAOCA and evaluation of hemodynamic changes after surgical repair.

### 9.7. Artificial Intelligence and Computational Modeling

AI-based approaches are being developed for automated coronary artery segmentation, anomaly detection, and risk prediction from CTA datasets. Computational fluid dynamics modeling may enable patient-specific simulation of coronary hemodynamics under exercise conditions, potentially improving risk stratification for AAOCA. Three-dimensional printing and virtual procedural planning from CT and CMR data may improve surgical approach planning in complex coronary anomalies [37].

### 9.8. Need for Multicenter Registries and Standardized Reporting

Given the low prevalence of individual coronary anomalies and the low event rates, multicenter registries with standardized reporting are essential to generate sufficient data for evidence-based management. The Congenital Heart Surgeons’ Society AAOCA Registry and similar initiatives represent important steps toward this goal. Standardized nomenclature and classification systems are needed to facilitate data aggregation across centers [30].

### 9.9. Genetic Basis of Coronary Anomalies

Emerging evidence suggests a potential genetic etiology for some coronary anomalies, with familial clustering reported in AAOCA. Screening first-degree relatives with echocardiography has been suggested but is not yet standardized [12]. Further research into the genetic and developmental mechanisms underlying coronary anomalies may identify at-risk individuals and inform screening strategies [11].

### 9.10. Future Research Directions

Despite evolving knowledge, significant gaps remain in understanding the natural history, optimal diagnosis, and management of coronary anomalies in adults with congenital heart disease. Priority areas for future research include:

- Establishment of international prospective registries capturing morbidity, mortality, and quality of life outcomes to stratify risk and evaluate interventions.
- Randomized trials comparing conservative versus surgical management in subgroups such as asymptomatic AAOCA patients with and without high-risk features.
- Evaluation of novel imaging modalities and invasive physiological techniques in large multicenter cohorts to standardize diagnostic algorithms.
- Development and validation of AI-based predictive models incorporating multimodal data to assist clinical decision-making.
- Investigation into patient-reported outcomes and long-term functional status to inform holistic care approaches.

Collaboration across specialist centers, leveraging advances in technology and data science, will be crucial to address these challenges and improve patient outcomes.

## 10. Conclusions

Coronary artery anomalies in ACHD represent a complex and evolving clinical challenge that spans the full spectrum from incidental benign variants to life-threatening conditions. The growing ACHD population, the maturation of the arterial switch generation, and advances in multimodality imaging have brought these anomalies into sharper clinical focus. A systematic approach integrating anatomic characterization (primarily by CTA), functional assessment (by stress imaging), and individualized risk stratification is essential for optimal management.

Key principles for the practicing clinician include: (1) awareness of the specific coronary anomalies associated with each congenital heart defect and their implications for surgical planning and reoperation; (2) routine pre-operative coronary imaging before any intervention on the right ventricular outflow tract or aortic root in repaired CHD; (3) a benchmark assessment of coronary anatomy in adults after the arterial switch operation; (4) shared decision-making for the management of asymptomatic patients with AAOCA, integrating anatomic risk features, physiologic testing, patient age, and guideline recommendations; and (5) lifelong surveillance for coronary complications in patients who have undergone operations involving coronary manipulation or transfer.

Important knowledge gaps remain, including the natural history of AAOCA in adults, the long-term coronary fate of the arterial switch generation, the optimal management of asymptomatic anomalous right coronary artery, and the role of emerging technologies such as CT-FFR, photon-counting CT, and 4D flow MRI. Multicenter registries, prospective outcome studies, and continued collaboration between ACHD cardiologists, cardiac surgeons, and imaging specialists will be essential to advance the field and improve outcomes for this growing patient population.

## Figures and Tables

**Table 1:** Classification of Native/Isolated Congenital Coronary Artery Anomalies.

Category	Anomaly Type	Subtypes/Variants	Proximal Course (If Applicable)	Prevalence	Hemodynamic Significance	Pathophysiology of Ischemia	Clinical Relevance
**Anomalies of Origin and Course**	AAOCA from wrong sinus of Valsalva	L-AAOCA from R sinus; R-AAOCA from L sinus; Cx from R sinus; LAD from R sinus; Origin from NCS	Interarterial (between aorta and PA); Retroaortic; Prepulmonic; Trans-septal (subpulmonic)	0.2–0.5% (autopsy); 1–5% (imaging)	High (interarterial/intramural); Low (retroaortic, prepulmonic, trans-septal)	Flap-like closure of slit-like orifice; acute angle takeoff with kinking; compression of intramural segment by aortic commissure; compression between great vessels during exercise; coronary spasm from endothelial injury	Second most common cause of SCD in young athletes; surgical repair indicated for high-risk features (interarterial course, intramural segment, slit-like orifice, acute angle takeoff, proximal hypoplasia, dominant anomalous vessel)
	ALCAPA	Anomalous LCA from PA	N/A	~1:300,000 live births	High	Coronary steal phenomenon with reversal of flow into low-pressure PA; myocardial ischemia from inadequate perfusion	Fatal in infancy without repair; adult survivors (“adult type”) with well-developed collaterals present with dyspnea, angina, HF, ventricular arrhythmias, or SCD; majority are women; surgical repair to establish dual-coronary system
	ARCAPA	Anomalous RCA from PA	N/A	Rarer than ALCAPA	High	Similar steal phenomenon as ALCAPA but typically better tolerated due to RCA territory	May present with ischemia, arrhythmias, or SCD; surgical repair recommended
	Single coronary artery	Single ostium supplying entire myocardium; variable branching patterns	Branches may follow interarterial, retroaortic, prepulmonic, or trans-septal courses	0.024–0.066%	Variable (depends on course of branches)	Ischemia if any branch traverses between great vessels	Risk determined by course of branches relative to great vessels; important for surgical planning
	High takeoff/Ectopic ostial location	Ostium above STJ; ostium in ascending aorta	N/A	Uncommon	Usually low	Generally does not cause ischemia	Important during cardiac surgery, aortic cannulation, aortotomy, and coronary angiography; risk of inadvertent injury
	Absent left main trunk	Separate ostia of LAD and Cx arising directly from left sinus	N/A	0.4–0.7%	None	N/A	Benign variant; important to recognize during angiography and surgery to avoid misdiagnosis
**Anomalies of Intrinsic Anatomy**	Myocardial bridging	Superficial (<2 mm depth); Deep (≥2 mm depth); most commonly mid-LAD	N/A	0.5–2.5% (angiography); 15–85% (autopsy)	Usually low; High if deep and/or long segment, especially with LVH or HCM	Systolic compression of tunneled segment; delayed diastolic relaxation; endothelial dysfunction; accelerated proximal atherosclerosis	Symptomatic ischemia possible, especially with LVH, HCM, or tachycardia; surgical myotomy or CABG for refractory cases; stenting generally not recommended
	Congenital coronary stenosis	Isolated ostial stenosis; ostial stenosis associated with supravalvar AS (Williams syndrome)	N/A	Rare	Variable; High if severe	Fixed obstruction to coronary flow; may be progressive	May require surgical coronary osteoplasty; detection can be difficult even with catheterization; often addressed concomitantly with supravalvar AS repair
	Coronary ectasia	Diffuse or segmental dilation; characteristic of Williams syndrome	N/A	Rare (isolated); common in supravalvar AS	Variable	Exposure of coronary arteries to elevated pressures proximal to supravalvar obstruction; turbulent flow and potential thrombosis	Associated with supravalvar AS; may coexist with ostial stenosis; surveillance for progressive dilation
	Congenital coronary aneurysm	Saccular or fusiform dilation (distinct from acquired/Kawasaki)	N/A	Very rare	Variable; High if large with risk of thrombosis or rupture	Turbulent flow; stasis and thrombosis; distal embolization	Risk of thromboembolism, rupture; may require surgical repair or anticoagulation
**Anomalies of Termination**	Coronary artery fistulae (congenital)	RCA origin (60%); LCA origin (35%); bilateral (5%); Drainage to RV (39%), RA (33%), PA (20%), coronary sinus, SVC, or LA	N/A	0.1–0.2% (angiography)	High if large shunt; Low if small	Coronary steal with diversion of flow away from myocardium; volume overload of receiving chamber; progressive dilation of feeding artery	Closure indicated if symptomatic or RV enlargement from significant L-to-R shunt; transcatheter closure is first-line for selected cases (90% acute success); surgical options include epicardial ligation, trans-arterial closure, transcardiac closure, arterioplasty; recanalization rate ~15%; postoperative MI risk ~11–18%; follow-up imaging recommended

**Table 2:** Classification by Hemodynamic Significance.

Classification	Anomalies Included	Key Features
**Hemodynamically significant (abnormal myocardial perfusion; risk of ischemia or SCD)**	AAOCA with interarterial course; AAOCA with intramural segment; ALCAPA; ARCAPA; Large coronary fistulae with significant shunting; Symptomatic deep myocardial bridging; Congenital coronary stenosis (severe)	Require comprehensive evaluation with anatomic imaging (CTA) and functional assessment (stress testing); surgical or interventional management often indicated
**Hemodynamically insignificant (benign variants)**	Retroaortic Cx; Separate ostia of LAD and Cx; Prepulmonic course; Trans-septal course; High takeoff without associated pathology; Small coronary fistulae; Superficial myocardial bridging (asymptomatic)	Generally require no intervention; important to recognize to avoid misdiagnosis and for surgical planning

**Table 3:** Coronary Artery Anomalies Associated with Specific Congenital Heart Defects.

Congenital Heart Defect	Common Coronary Anomalies	Prevalence	Clinical Significance	Surgical Planning Implications
**Tetralogy of Fallot**	LAD from RCA crossing RVOT; Dual LAD; Single coronary; Large conus arteries; Coronary AV fistulae	4–14% (anomalous coronary); ~10.3% (any vessel crossing RVOT)	Risk of injury during RVOT surgery	Mandatory coronary imaging before PVR or RVOT intervention (Class I)
**d-TGA**	Variable patterns; Single coronary; Intramural coronary; Inverted patterns	Highly variable	Intramural coronary = independent predictor of mortality (HR 5.2) and reintervention (HR 33.9)	Coronary pattern dictates transfer technique at ASO
**ccTGA**	Reversed coronary pattern; Short main stem with early branching; Single coronary from R-facing sinus	Common (reversed pattern is the norm)	Coronaries may be entrapped in RV fat/muscle	Important for double-switch or Senning-Rastelli planning
**DORV**	Clockwise rotation of ostia; LAD from RCA crossing RVOT	Variable	Similar to TOF when anatomy overlaps	Coronary imaging essential before RVOT procedures
**Truncus Arteriosus**	High takeoff above sinuses; Single coronary origins; Anomalous courses	Common	Risk of transection during aortotomy	Identify high takeoff before truncal valve surgery
**PA/IVS**	RV-coronary fistulae (sinusoids); Coronary stenoses; RV-dependent coronary circulation	Common in severe forms	RV decompression may be lethal if RV-dependent	Angiographic assessment of coronary circulation mandatory
**Supravalvar AS (Williams)**	Coronary ectasia	Characteristic	Exposure to elevated proximal pressures	Assess coronary anatomy before aortic surgery
**BAV**	AAOCA (nearly double prevalence vs. TAV)	~2× general population	May coexist with aortopathy	Screen for AAOCA during pre-op evaluation for AVR

Abbreviations: LAD, left anterior descending artery; RCA, right coronary artery; RVOT, right ventricular outflow tract; AV, arteriovenous; PVR, pulmonary valve replacement; d-TGA, dextro-transposition of the great arteries; HR, hazard ratio; ASO, arterial switch operation; ccTGA, congenitally corrected transposition of the great arteries; RV, right ventricle; DORV, double outlet right ventricle; TOF, tetralogy of Fallot; PA/IVS, pulmonary atresia with intact ventricular septum; AS, aortic stenosis; BAV, bicuspid aortic valve; AAOCA, anomalous aortic origin of a coronary artery; TAV, tricuspid aortic valve; AVR, aortic valve replacement.

**Table 4:** Postoperative (Iatrogenic) Coronary Complications.

Operation	Coronary Complication	Reported Incidence	Risk Factors	Recommended Surveillance	Management Options
**ASO**	Coronary occlusion/stenosis	2–7% asymptomatic occlusion; 2–2.8% reintervention	Intramural coronary (HR 33.9 for reintervention); Acute LCA angulation; Clockwise ostial rotation	Benchmark CTA or catheter angiography in adults; Annual echo; CMR every 3–5 years	Button revision, ostioplasty, interposition graft, CABG
**ASO**	Neo-PA compression of coronary	Uncommon	LeCompte maneuver; Anterior coronary button position	Cross-sectional imaging if symptoms	PA translocation, coronary revision
**ASO**	Neoaortic root dilation	18% (z-score ≥ 4)	Typically beyond first decade	Serial echo and CMR	Aortic root replacement if progressive
**Ross Procedure**	Coronary button aneurysm	Uncommon	Unsupported autograft tissue at systemic pressure	Serial cross-sectional imaging (CTA/CMR)	Autograft reinforcement, reoperation
**Ross Procedure**	Coronary kinking from autograft dilation	Uncommon	Progressive autograft dilation with cephalad/anterior button displacement	Serial echo and CMR	Reoperation with coronary revision
**ALCAPA Repair**	Residual ischemia, graft stenosis, myocardial fibrosis	Variable	Pre-operative ischemic injury; Takeuchi baffle complications	Periodic stress testing and cross-sectional imaging	Balloon dilation, stenting, reoperation
**TOF Repair**	Iatrogenic coronary injury during RVOT reconstruction	Rare	Unrecognized anomalous LAD crossing RVOT	Pre-operative CTA before redo sternotomy or PVR	CABG, coronary revision
**Redo Sternotomy (any CHD)**	Coronary laceration during sternal re-entry	Rare	Coronary adherent to posterior sternum; Conduit adherent to sternum	Pre-operative CT to define coronary-sternal relationship	Emergent CABG, femoral cannulation

Abbreviations: ASO, arterial switch operation; HR, hazard ratio; LCA, left coronary artery; CTA, computed tomography angiography; CMR, cardiac magnetic resonance; CABG, coronary artery bypass grafting; Neo-PA, neopulmonary artery; PA, pulmonary artery; ALCAPA, anomalous left coronary artery from the pulmonary artery; TOF, tetralogy of Fallot; RVOT, right ventricular outflow tract; LAD, left anterior descending artery; PVR, pulmonary valve replacement; CHD, congenital heart disease; CT, computed tomography.

**Table 5:** Comparison of Imaging Modalities for Coronary Assessment in ACHD.

Feature	Coronary CTA	CMR	Echocardiography	Invasive Angiography
**Spatial Resolution**	Highest (~0.3–0.5 mm)	Moderate (~1–1.5 mm)	Variable (operator-dependent)	High (~0.2 mm)
**Radiation Exposure**	Yes (1–5 mSv)	None	None	Yes (5–15 mSv)
**Coronary Origin/Course**	Excellent (gold standard)	Good (~94% success for origins)	Limited in adults	Good (2D only)
**Intramural Segment Detection**	Excellent	Good	Poor	Limited
**Ostial Morphology**	Excellent	Moderate	Poor	Moderate
**3D Anatomic Relationships**	Excellent	Good	Limited	Poor (2D projections)
**Myocardial Perfusion**	Limited (CT-FFR emerging)	Excellent (stress perfusion)	Moderate (stress echo)	FFR available
**Myocardial Fibrosis/Scar**	Limited	Excellent (LGE)	Not available	Not available
**Ventricular Function**	Good	Excellent (reference standard)	Good	Limited
**Atherosclerosis Assessment**	Excellent	Poor	Not available	Excellent
**Pre-operative Planning**	Excellent (sternal relationships)	Good	Limited	Limited
**Guideline Recommendation (2025 ACR)**	“Usually appropriate”—initial test	“Usually appropriate”—alternative	First-line screening	When stenosis or hemodynamic data needed
**ACHD-Specific Advantages**	Conduit/sternal relationships; Coronary-RVOT relationship in TOF	No radiation (ideal for serial follow-up); Combined ventricular/valvular assessment	Widely available; Bedside; Functional baseline	IVUS/FFR capability; Interventional potential
**Key Limitations**	Radiation; Iodinated contrast; Heart rate dependence	Lower coronary spatial resolution; Longer acquisition; Device contraindications	Limited acoustic windows in adults; Operator-dependent	Invasive; 2D only; Contrast/radiation

Abbreviations: CTA, computed tomography angiography; CMR, cardiac magnetic resonance; mSv, millisievert; 2D, two-dimensional; 3D, three-dimensional; CT-FFR, computed tomography–derived fractional flow reserve; FFR, fractional flow reserve; LGE, late gadolinium enhancement; ACR, American College of Radiology; RVOT, right ventricular outflow tract; TOF, tetralogy of Fallot; IVUS, intravascular ultrasound; ACHD, adult congenital heart disease.

**Table 6:** Risk Stratification Framework for AAOCA in Adults.

Risk Category	Anatomic Features	Physiologic Findings	Patient Factors	Recommended Management
**High**	Interarterial course WITH intramural segment; Slit-like/fish-mouth orifice; Acute angle takeoff (45°); Proximal vessel hypoplasia; Dominant anomalous vessel	Inducible ischemia on stress testing; Reduced FFR; Regional wall motion abnormalities	Age 35 years; Competitive athlete; Symptoms (angina, syncope, aborted SCD)	Surgical repair recommended (Class I if symptomatic; Class IIa if asymptomatic L-AAOCA with high-risk features); Activity restriction until repair
**Intermediate**	Interarterial course WITHOUT intramural segment; Equivocal ostial morphology; R-AAOCA with interarterial course	Equivocal or negative stress test; No inducible ischemia but high-risk anatomy	Asymptomatic; Age 35–50 years; Non-dominant anomalous vessel	Shared decision-making: Surgery vs. observation with surveillance; Serial stress testing; Activity counseling (Class IIb)
**Low**	Retroaortic course; Prepulmonic course; Separate LAD/Cx ostia; High takeoff without other pathology	No ischemia; Normal stress test; Normal ventricular function	Asymptomatic; Any age; Incidental finding	Reassurance; No routine follow-up imaging required; No activity restriction

Abbreviations: FFR, fractional flow reserve; SCD, sudden cardiac death; L-AAOCA, anomalous left coronary artery from the right aortic sinus; R-AAOCA, anomalous right coronary artery from the left aortic sinus; LAD, left anterior descending artery; Cx, circumflex artery.

**Table 7:** AHA/ACC Versus ESC Guideline Recommendations for Coronary Anomalies.

Clinical Scenario	AHA/ACC 2018 (COR/LOE)	ACC/AHA 2025 (COR/LOE)	ESC 2020 (COR/LOE)	Key Divergence
**Symptomatic AAOCA with ischemia (either coronary)**	Class I/B	Class I/B	Class I/C	Broad agreement; ESC requires positive stress test OR high-risk anatomy
**Asymptomatic L-AAOCA, no ischemia**	Class IIa/C	Class IIa/C (high-risk features); Class IIb (without)	Class IIa (if 35 y); Class III (no ischemia, no high-risk features)	AHA/ACC more aggressive; ESC recommends against surgery without ischemia
**Asymptomatic R-AAOCA, no ischemia, no high-risk features**	Class IIb/C (surgery or observation)	Class IIb/C (shared decision-making)	Class III (surgery not recommended)	Major divergence: AHA/ACC = equipoise; ESC = against surgery
**AAOCA with ventricular arrhythmias**	Class IIa/C	Class IIa/C	Not specifically addressed	AHA/ACC supports repair to mitigate arrhythmia recurrence
**ALCAPA (symptomatic or asymptomatic)**	Class I/B	Class I/B	Class I/C	Agreement: surgical repair regardless of symptoms
**ARCAPA with ischemia or ventricular dysfunction**	Class IIa/C	Class IIa/C	Class IIa/C	Agreement: repair reasonable
**Coronary fistula—symptomatic or large with RV enlargement**	Class I/C	Class I/C	Class I/C	Agreement: closure indicated
**Diagnostic workup—initial imaging**	CTA or CMR (Class I/C)	CTA preferred (Class I)	Nonpharmacologic functional imaging (Class I/C)	ESC emphasizes functional imaging first; AHA/ACC emphasizes anatomic imaging
**Stress testing modality**	Exercise or dobutamine preferred	Exercise or dobutamine preferred over vasodilator	Nonpharmacologic stress recommended	Agreement on exercise/dobutamine; ESC less specific

Abbreviations: COR, class of recommendation; LOE, level of evidence; AAOCA, anomalous aortic origin of a coronary artery; L-AAOCA, anomalous left coronary artery from the right aortic sinus; R-AAOCA, anomalous right coronary artery from the left aortic sinus; ALCAPA, anomalous left coronary artery from the pulmonary artery; ARCAPA, anomalous right coronary artery from the pulmonary artery; RV, right ventricle; CTA, computed tomography angiography; CMR, cardiac magnetic resonance; AHA, American Heart Association; ACC, American College of Cardiology; ESC, European Society of Cardiology.

## Data Availability

Not applicable. No original datasets were generated or analyzed during the current review. All data discussed are available in the cited published literature.

## Abbreviations

AAOCA	Anomalous aortic origin of the coronary artery
ACHD	Adults with congenital heart disease
ALCAPA	Anomalous left coronary artery origin from the pulmonary artery
ARCAPA	Anomalous right coronary artery origin from the pulmonary artery
ASO	Arterial switch operation
CA	Coronary artery
CHD	Congenital heart disease
CMR	Cardiac magnetic resonance imaging
CTA	Computed tomographic angiography
DORV	Double outlet right ventricle
FFR	Fractional flow reserve
IVUS	Intravascular ultrasound
LAD	Left anterior descending coronary artery
RVOT	Right ventricular outflow tract
SCD	Sudden cardiac death
TOF	Tetralogy of Fallot
